# Historical comparison of gender inequality in scientific careers across countries and disciplines

**DOI:** 10.1073/pnas.1914221117

**Published:** 2020-02-18

**Authors:** Junming Huang, Alexander J. Gates, Roberta Sinatra, Albert-László Barabási

**Affiliations:** ^a^Network Science Institute and Department of Physics, Northeastern University, Boston, MA 02115;; ^b^CompleX Lab, School of Computer Science and Engineering, University of Electronic Science and Technology of China, Chengdu 611731, China;; ^c^Paul and Marcia Wythes Center on Contemporary China, Princeton University, Princeton, NJ 08540;; ^d^Department of Computer Science, IT University of Copenhagen, 2300 Copenhagen, Denmark;; ^e^ISI Foundation, 10126 Turin, Italy;; ^f^Channing Division of Network Medicine, Brigham and Women’s Hospital, Harvard Medical School, Boston, MA 02115;; ^g^Department of Medicine, Brigham and Women’s Hospital, Harvard Medical School, Boston, MA 02115;; ^h^Department of Network and Data Science, Central European University, 1051 Budapest, Hungary

**Keywords:** gender inequality, science of science, STEM, scientific careers

## Abstract

There is extensive, yet fragmented, evidence of gender differences in academia suggesting that women are underrepresented in most scientific disciplines and publish fewer articles throughout a career, and their work acquires fewer citations. Here, we offer a comprehensive picture of longitudinal gender differences in performance through a bibliometric analysis of academic publishing careers by reconstructing the complete publication history of over 1.5 million gender-identified authors whose publishing career ended between 1955 and 2010, covering 83 countries and 13 disciplines. We find that, paradoxically, the increase of participation of women in science over the past 60 years was accompanied by an increase of gender differences in both productivity and impact. Most surprisingly, though, we uncover two gender invariants, finding that men and women publish at a comparable annual rate and have equivalent career-wise impact for the same size body of work. Finally, we demonstrate that differences in publishing career lengths and dropout rates explain a large portion of the reported career-wise differences in productivity and impact, although productivity differences still remain. This comprehensive picture of gender inequality in academia can help rephrase the conversation around the sustainability of women’s careers in academia, with important consequences for institutions and policy makers.

## Significance

Empirical evidence suggests significant gender differences in the total productivity and impact of academic careers across science, technology, engineering, and mathematics (STEM) fields. Paradoxically, the increase in the number of women academics over the past 60 years has increased these gender differences. Yet, we find that men and women publish a comparable number of papers per year and have equivalent career-wise impact for the same total number of publications. This suggests the productivity and impact of gender differences are explained by different publishing career lengths and dropout rates. This comprehensive picture of gender inequality in academic publishing can help rephrase the conversation around the sustainability of women’s careers in academia, with important consequences for institutions and policy makers.

Gender differences in academia, captured by disparities in the number of female and male authors, their productivity, citations, recognition, and salary, are well documented across all disciplines and countries (1–8). The epitome of gender difference is the “productivity puzzle” (9–13)—the persistent evidence that men publish more than women over the course of their career, which has inspired a plethora of possible explanations (14–16), from differences in family responsibilities (17–19), to career absences (20), resource allocation (21), the role of peer review (22), collaboration (23, 24), role stereotypes (25), academic rank (26), specialization (27), and work climate (28). The persistence of these gender differences could perpetuate the naive interpretation that the research programs of female and male scientists are not equivalent. However, such simplistic reading of the data dismisses increasing evidence that systemic barriers impede the female academic. Indeed, the deep interrelatedness of these factors has limited our ability to differentiate the causes from the consequences of the productivity puzzle, complicating the scientific community’s ability to enact effective policies to address it.

A key methodological obstacle has been the difficulty to reconstruct full publishing careers for scientists of both genders across the diverse academic population. Consequently, much of the available evidence on gender differences is based on case studies limited to subsets of active scientists in specific countries, disciplines, or institutions, making it difficult to compare and generalize the finding to all of science. A further complication arises from the heavy-tailed nature of academia: a disproportionately small number of authors produce a large fraction of the publications and receive the majority of the citations (29), an effect that is exacerbated in small sample sizes (30). To truly understand the roots of the gender inequality, we need to survey the whole longitudinal, disciplinary, and geographical landscape, which is possible only if we capture complete publishing careers for all scientists across disciplinary and national boundaries.

Here, we reconstructed the full publishing career of 7,863,861 scientists from their publication record in the Web of Science (WoS) database between 1900 and 2016. By deploying a state-of-the-art method for gender identification (*SI Appendix*, section S2.E), we identified the gender of over 3 million authors (856,889 female and 2,146,926 male) spanning 83 countries and 13 major disciplines (*SI Appendix*, section S2). We then focused on 1,523,002 scientists (412,808 female and 1,110,194 male) whose publishing careers ended between 1955 and 2010 (*SI Appendix*, sections S1 and S2.H), allowing us to systematically compare complete male and female careers. This extensive sample covers 33% of all papers published between 1955 and 2010 but due to methodological limitations, systematically lacks authors from China, Japan, Korea, Brazil, Malaysia, and Singapore (*SI Appendix*, section S2). To demonstrate the robustness of our findings to database bias and author disambiguation errors, we independently replicated our results in two additional datasets: the Microsoft Academic Graph (MAG) (31) and the Digital Bibliography & Library Project (DBLP), each using different criteria for publication inclusion and methodologies for career reconstruction (*SI Appendix*, sections S1 and S6). Our focus on bibliometric data limits our analysis to publishing careers and is unable to capture the career dynamics of teaching, administrative, industrial, or government related research activities. Nevertheless, our efforts constitute an extensive attempt to quantify gender inequality in science, technology, engineering, and mathematics (STEM) publications and citations, offering a longitudinal, career-wise perspective across national and disciplinary boundaries.

## The Increasing and Persistent Gender Gap

**Fig. 1.**
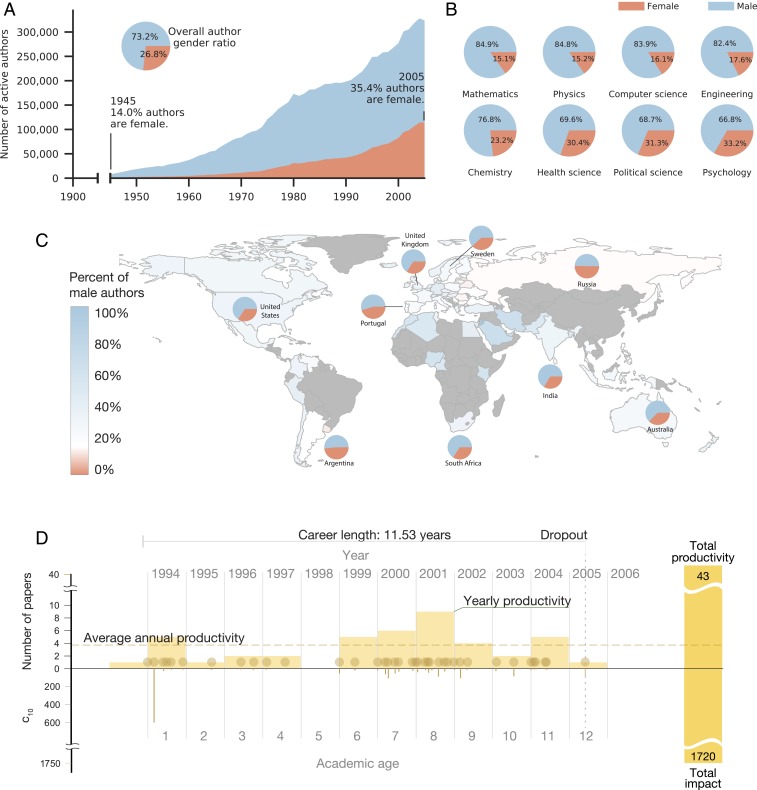
Gender imbalance since 1955. (*A*) The number of active female (orange) and male (blue) authors over time and the total proportions of authors (*Inset*). (*B* and *C*) The proportion of female authors in several disciplines (*B*) and countries (*C*); for the full list, see *SI Appendix*, Tables S3 and S4. (*D*) The academic publishing career of a scientist is characterized by his or her temporal publication record. For each publication, we identify the date (gold dot) and number of citations after 10 years $c_{10}$ (gold line, lower). The aggregation by year provides the yearly productivity (light gold bars), while the aggregation over the entire career yields the total productivity (solid yellow bar, right) and total impact (solid yellow bar, right). Career length is calculated as the time between the first and last publication, and the annual productivity (dashed gold line) represents the average yearly productivity. Authors drop out from our data when they published their last article.

Across all years and disciplines, women account for 27% of authors, a number that hides important trends: while in 1955 women represented only 12% of all active authors, that fraction steadily increased over the last century, reaching 35% by 2005 (Fig. 1*A*). Yet, these aggregate numbers hide considerable disciplinary differences, as the fraction of women is as low as 15% in math, physics, and computer science and reaches 33% in psychology (Fig. 1*B*). We also observe significant variations by country, finding that the proportion of female scientists can be as low as 28% in Germany and reaches parity with 50% in Russia (Fig. 1*C*).

**Fig. 2.**
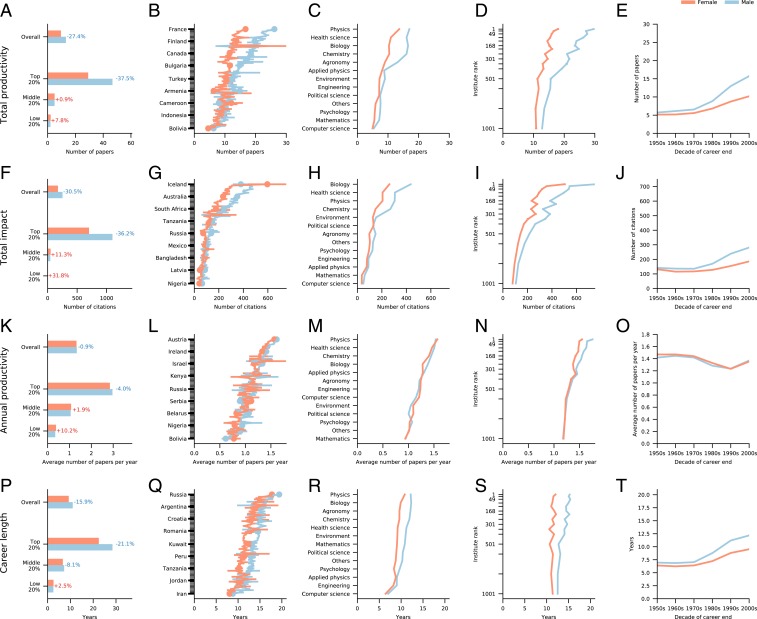
Gender gap in scientific publishing careers. The gender gap is quantified by the relative difference between the mean for male (blue) and female (orange) authors. In all cases the, relative gender differences are statistically significant, as established by the two-sided *t* test, with *P* values < $10^{-4}$, unless otherwise stated (see *SI Appendix*, section S4.A for test statistics). (*A*–*E*) Total productivity broken down by percentile (*A*), discipline (*B*), country (*C*), affiliation rank (*D*), and decade (*E*). The gender gap in productivity has been increasing from the 1950s to the 2000s. (*F*–*J*) Total impact subdivided by percentile (*F*), discipline (*G*), country (*H*), affiliation rank (*I*), and decade (*J*). (*K–O*) Annual productivity is nearly identical for male and female authors when subdivided by percentile (*K*), discipline (*L*), country (*M*), affiliation rank (*N*), and decade (*O*). (*P*–*T*) Career length broken down by percentile (*P*), discipline (*Q*), country (*R*), affiliation rank (*S*), and decade (*T*).

The low proportion of women actively publishing in STEM captures only one aspect of gender inequality. Equally important are the persistent productivity and impact differences between the genders (Fig. 1*D*). We find that while, on average, male scientists publish 13.2 papers during their career, female authors publish only 9.6, resulting in a 27% gender gap in total productivity (Fig. 2*A*). The difference is particularly pronounced among productive authors, as male authors in the top 20% productivity bracket publish 37% more papers than female authors (Fig. 2*A*). Interestingly, the gender gap disappears for median productive authors (middle 20%) and reverses for the authors in the bottom 20%. The gender gap in total productivity persists for all disciplines and almost all countries (Fig. 2 *B* and *C*). We also observe a large gender gap in total productivity for the highest-ranked affiliations (Fig. 2*D*) (determined from the 2019 Times Higher Education World University Rankings; *SI Appendix*, section S2.D).

We measure the total impact during an academic career by the number of citations accrued 10 years after publication ($c_{10}$) by each paper published during a career (Fig. 1*D*), after removing self-citations and rescaling to account for citation inflation (32–34) (*SI Appendix*, section S2.F). We find that male scientists receive 30% more citations for their publications than female scientists (Fig. 2*F*). Once again, the total impact difference is the largest for high-impact authors and reverses for median- and low-impact authors: male authors in the top 20% in career impact receive 36% more citations than their female counterparts. The disparity in impact persists in almost all countries and all disciplines (Fig. 1 *G* and *H*), and can be found, to a lesser extent, across all affiliations regardless of affiliation rank (Fig. 1*I*).

Paradoxically, the gradual increase in the fraction of women in science (5) (Fig. 1*A*) is accompanied by a steady increase in both the productivity and impact gender gaps (Fig. 2 *E* and *J*). The gender gap in total productivity rose from near 10% in the 1950s to a strong bias toward male productivity (35% gap) in the 2000s. The gender gap in total impact actually switches from slightly more female impact in the 1950s to a 34% gap favoring male authors in the same time frame. These observations disrupt the conventional wisdom that academia can achieve gender equality simply by increasing the number of participating female authors.

In summary, despite recent attempts to level the playing field, men continue to outnumber women 2 to 1 in the scientific workforce and, on average, have more productive careers and accumulate more impact. These results confirm, using a unified methodology spanning most of science, previous observations in specific disciplines and countries (2, 9, 11, 12, 16, 35–38) and support in a quantitative manner the perception that global gender differences in academia is a universal phenomenon persisting in every STEM discipline and in most geographic regions. Moreover, we find that the gender gaps in productivity and impact have increased significantly over the last 60 years. The universality of the phenomenon prompts us to ask: What characteristics of academic careers drive the observed gender-based differences in total productivity and impact?

## Annual Productivity and Career Length

As total productivity and impact over a career represent a convolution of annual productivity and publishing career length, to identify the roots of the gender gap, we must separate these two factors. Traditionally, the difficulty of reconstructing full publishing careers has limited the study of annual productivity to a small subset of authors or to career patterns observable during a fixed time frame (39–46). Access to the full publishing career data allows us to decompose each author’s total productivity into his or her annual productivity and career length, defined as the time span between a scientist’s first and last publication (Fig. 1*D* and *SI Appendix*, section S3). We find that the annual productivity differences between men and women are negligible: female authors publish, on average, 1.33 papers per year, while male authors publish, on average, 1.32 papers per year, a difference, that while statistically significant, is considerably smaller than other gender disparities (0.9%, *P* value $<10^{-9}$; Fig. 2*K*). This result is observed in all countries and disciplines (Fig. 2 *L* and *M*), and we replicated it in all three datasets (*SI Appendix*, section S6). The gender difference in annual productivity is small even among the most productive authors (4% for the top 20%) and is reversed for authors of median and low productivity.

The average annual productivity of scientists has slightly decreased over time; yet, there is consistently no fundamental difference between the genders (Fig. 2*O*). In other words, when it comes to the number of publications per year, female and male authors are largely indistinguishable, representing the first gender invariant quantity in performance metrics. As we show next, this invariant, our key result, helps us probe the possible roots of the observed gender gaps.

**Fig. 3.**
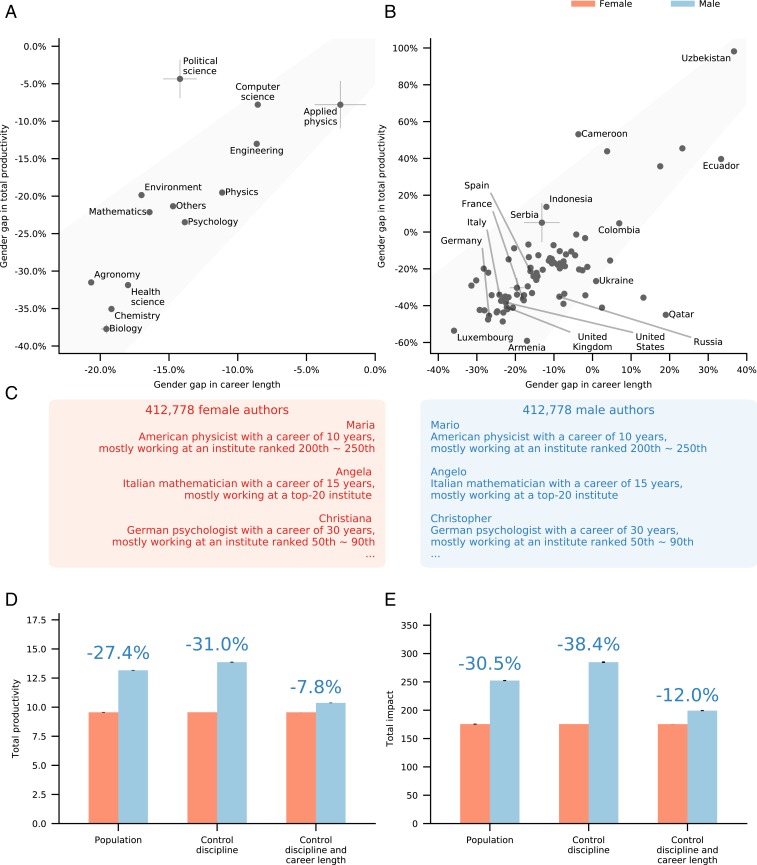
Controlling for career length. (*A* and *B*) The gender gap in career length strongly correlates with the gender gap in productivity across disciplines (Pearson correlation, 0.80) (*A*) and countries (Pearson correlation, 0.56) (*B*). A gender gap of 0.0% indicates gender equality, while negative gaps indicate the career length or productivity is greater for male careers, and positive gaps indicate the feature is greater for female careers. (*C*) In a matching experiment, equal samples are constructed by matching every female author with a male author having an identical discipline, country, and career length. (*D*) The average productivity provided by the matching experiment for career length compared to the population; the gender gap is reduced from 27.4% in the population to 7.8% in the matched samples. (*E*) The average impact provided by the matching experiment for career length compared to the original unmatched sample. Where visible, error bars denote 1 SD.

The comparable annual productivity of male and female scientists suggests that the large gender gap in total career productivity is determined by differences in career length. To test if this is the case, we measured the career length (Fig. 1*D*) of each scientist in the database, finding that, on average, male authors reach an academic age of 11.0 years before ceasing to publish, while the average terminal academic age of female authors is only 9.3 years (Fig. 2*P*). This gap persists when authors are grouped by either discipline, country, or affiliation (Fig. 2 *Q*, *R*, and *S*) and has been increasing over the past 60 years (Fig. 2*T*). Taken together, Fig. 2 *K* and *T* suggests that a significant fraction of the variation in total productivity is rooted in variations in career lengths. This conclusion is supported by a strong correlation between the career-length gap and the career-wise productivity gap when we subdivide scientists by discipline (Fig. 3*A*; Pearson correlation, 0.80) and country (Fig. 3*B*; Pearson correlation, 0.58). In other words, this strong correlation implies that disciplines or countries with a large gender difference in the career length also have a large gender difference in total productivity, while those disciplines or countries with small gender differences in the career length also have a small gender difference in total productivity. For example, the gender gap in career length is smallest in applied physics (2.6%), as so is the gender gap in total productivity (7.8%). In contrast, in biology and chemistry, men have 19.2% longer careers on average, resulting in a total productivity gender gap that exceeds 35.1%.

Given the largely indistinguishable annual productivity patterns, we next ask how much of the total productivity and the total impact gender gaps observed above (Fig. 2 *A* and *F*) could be explained by the variation in career length. For this, we perform a matching experiment designed to eliminate the gender gaps in career length. In the first population, for each female scientist, we select a male scientist from the same discipline (Fig. 3*C* and *SI Appendix*, section S4.B). We then constructed a second matched population, as a subset of the first, in which each female scientist is matched to a male scientist from the same discipline and with exactly the same career length. In these career length-matched samples, the gender gap in total productivity reduces from 31.0 to 7.8% (Fig. 3*D*). Furthermore, the gender gap in the total impact is also reduced from 38.4 to 12.0% (Fig. 3*E*). By matching pairs of authors based on observable confounding variables, such as their discipline, we mitigate the influence of these variables on the gender gaps. More strenuous matching criteria controlling for country and affiliation rank do not greatly affect these results, although they limit us to much smaller matched populations (*SI Appendix*, section S4.B and Fig. S1). While matching cannot rule out that gender differences are influenced by unmatched variables that are unobserved here, the significant decrease in the productivity and impact gender gaps when we control for career length suggests that publication career length is a significant correlate of gender differences in academia.

**Fig. 4.**
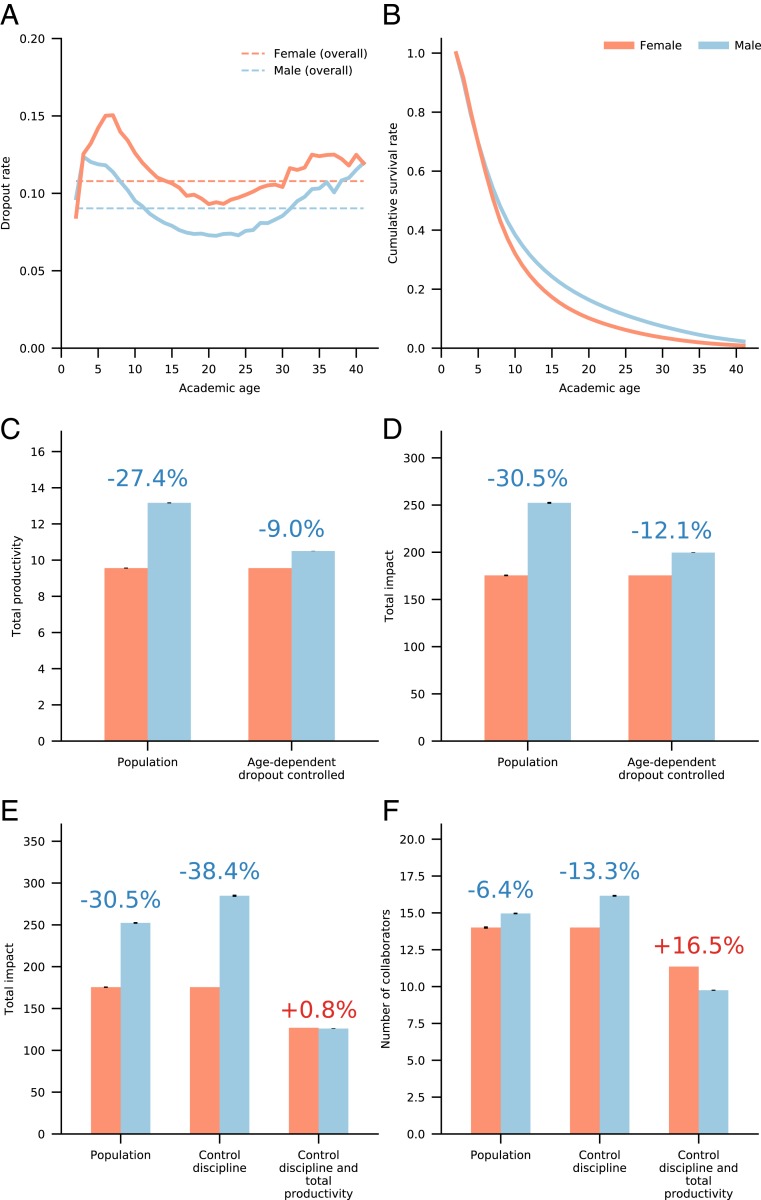
Author’s age-dependent dropout rate. (*A*) Dropout rate for male (blue) and female (orange) authors over their academic ages. (*B*) The cumulative survival rate for male and female authors over their academic ages. (*C* and *D*) The effect of controlling for the age-dependent dropout rate on the gender gaps in total productivity (*C*) and impact (*D*). (*E*) The total impact gap is eliminated in the matched sample based on total productivity. (*F*) The gender gap in the total number of collaborators is eliminated in the matched sample based on total productivity.

To address the factors governing the end of a publishing career, we calculated the dropout rate, defined as the yearly fraction of authors in the population who have just published their last paper (42, 47). We find that, on average, 9.0% of active male scientists stop publishing each year, while the yearly dropout rate for women is nearly 10.8% (Fig. 4*A*). In other words, each year, women scientists have a 19.5% higher risk to leave academia than male scientists, giving male authors a major cumulative advantage over time. Moreover, this observation demonstrates that the dropout gap is not limited to junior researchers but persists at similar rates throughout scientific careers.

The average causal effect of this differential attrition is demonstrated through a counterfactual experiment in which we shorten the careers of male authors to simulate dropout rates matching their female counterparts at the same career stage (Fig. 4 *C* and *D* and *SI Appendix*, section S4.F). We find that under similar dropout rates, the differences in total productivity and total impact reduce by roughly two-thirds, namely from 27.4 to 9.0% and from 30.5 to 12.1%, respectively. This result, combined with our previous matching experiment (Fig. 3 *D* and *E*), suggests that the difference in dropout rates is a key factor in the observed total productivity and impact differences, accounting for about 67% of the productivity and impact gaps. Yet, the differential dropout rates do not account for the whole effect, suggesting that auxiliary disruptive effects, from perception of talent to resource allocation (15, 21), may also play a potential role.

The reduction of the gender gaps in both total productivity and total impact by similar amounts suggests that total impact, being the summation over individual articles, may be primarily dependent on productivity (15). To test this hypothesis, we conducted another matching experiment in which we selected a male author from the same discipline and with exactly the same number of total publications as each female author (*SI Appendix*, section S4.D). In these matched samples, the gender gap in the total impact is completely eliminated, dropping from 38.4% in favor of male authors to 0.8% in favor of female authors (Fig. 4*E*). This reveals a second gender-invariant quantity—there is no discernible difference in impact between male and female scientists for the same size body of work. This second gender invariant reinforces our main finding that it is career-length differences that drive the total productivity gap, which consequently drives the impact gender gap in academia. Interestingly, controlling for productivity similarly flips the gender gap in the total number of collaborators throughout a career, from 13.3% in favor of male authors to 16% in favor of female authors (Fig. 4*F* and *SI Appendix*, section S4.E).

## Summary and Discussion

The reconstruction of full publishing careers of scientists allowed us to confirm the differences in total productivity and impact between female and male scientists across disciplines and countries since 1955. We showed that the gradual increase in the fraction of women in STEM was accompanied by an increase in the gender disparities in productivity and impact. It is particularly troubling that the gender gap is the most pronounced among the highly productive authors—those who train the new generations of scientists and serve as role models for them. Yet, we also found two gender invariants, revealing that active female and male scientists have largely indistinguishable yearly performance and receive a comparable number of citations for the same size body of work. These gender-invariant quantities allowed us to show that a large portion of the observed gender gaps are rooted in gender-specific dropout rates and the subsequent gender gaps in publishing career length and total productivity. This finding suggests that we must rephrase the conversation about gender inequality around the sustainability of woman’s careers in academia, with important administrative and policy implications (16, 37, 48–53).

It is often argued that in order to reduce the gender gap, the scientific community must make efforts to nurture junior female researchers. We find, however, that the academic system is losing women at a higher rate at every stage of their careers, suggesting that focusing on junior scientists alone may not be sufficient to reduce the observed career-wise gender imbalance. The cumulative impact of this career-wide effect dramatically increases the gender disparity for senior mentors in academia, perpetuating the cycle of lower retention and advancement of female faculty (10, 53–55).

Our focus on closed careers limited our study to careers that ended by 2010, eliminating currently active careers. Therefore, further work is needed to detect the impact of recent efforts by many institutions and funding agencies to support the participation of women and minorities (41, 56). Our analysis of all careers and the factors that dominate the gender gap could offer a baseline for such experimental studies in the future. Due to the reliability of gender disambiguation, we were also unable to assess author gender for China, Japan, Korea, Brazil, Malaysia, and Singapore, whose inclusion would provide a more comprehensive global perspective of gender differences in science. Since scientists from these countries significantly increased their contributions to the global scientific discourse, there is a pressing need for future work to develop more accurate gender identification methodologies. Despite these limitations, our work suggests the importance of temporal controls for studying academic careers and, in particular, gender inequality in academia.

It is important to emphasize that the end of a publishing career does not always imply an end of an academic career; authors who stopped publishing often retain teaching or administrative duties or conduct productive research in industry or governmental positions, with less pressure to communicate their findings through research publications. Scientific publications represent only one of the possible academic outputs; in some academic disciplines, books and patents are equally important, and all three of our data sources (WoS, MAG, and DBLP) tend to overrepresent STEM and English language publications (57), thereby possibly biasing our analysis. Furthermore, our bibliometric approach can draw deep insight into the large-scale statistical patterns reflecting gender differences, and yet we cannot observe and test potential variation in the organizational context and resources available to individual researchers (13, 58). However, our results do suggest important consequences for the organizational structures within academic departments. Namely, we find that a key component of the gender gaps in productivity and impact may not be rooted in gender-specific processes through which academics conduct research and contribute publications but by the gender-specific sustainability of that effort over the course of an entire academic career.

### Data and Code Availability.

The DBLP and MAG are publicly available from their source websites (*SI Appendix*). Other related and relevant data and code are available from the corresponding author upon request.

## Supplementary Material

Supplementary File
