# Supplementary material for: Historical comparison of gender inequality in scientific careers across countries and disciplines
Source: Proc Natl Acad Sci U S A. 2020 Feb 18;117(9):4609–16. doi: 10.1073/pnas.1914221117 (PMC7060730; doi:10.1073/pnas.1914221117)
Supplement: Supplementary File [file pnas.1914221117.sapp.pdf]

Supplementary Information for

Historical comparison of gender inequality in scientific careers across countries and disciplines

Junming Huang, Alexander J. Gates, Roberta Sinatra, Albert-László Barabási

Albert-László Barabási.
E-mail: a.barabasi@northeastern.edu

This PDF file includes:

Supplementary text
Figs. S1 to S17
Tables S1 to S8
References for SI reference citations

www.pnas.org/cgi/doi/10.1073/pnas.1914221117

Supporting Information Text

1. Data sets

A. Web of Science. The primary source of publication data for this project is the Clarivate Analytics' Web of Science Core Collection (WoS) database, covering the Science Citation Index Expanded and the Social Sciences Citation Index. In total, we consider the publication history of 7,863,861 authors who contributed a total of 101,961,318 authorships to 53,788,499 publications. Additionally, we extracted the citation history for all publications, resulting in 694,439,758 citation relationships.

The WoS dataset assigns each article to at least one scientific discipline in a three-layer hierarchy of 153 disciplines. For example, a paper is assigned to "Science & Technology" (top layer), "Life Sciences & Biomedicine" (middle layer) and "Biophysics" (leaf layer). The assignment is primarily based on each publication's journal information, but a select few multidisciplinary journals (e.g. *Nature* and *Science*) provide article-specific categories. For our purposes, the 153 disciplines in the leaf layer are too fine grained, while the other two layers do not provide a detailed enough classification. Therefore, we grouped the leaf layer categories into a coarser partition as described in Section 2.G.

B. Microsoft Academic Graph. The Microsoft Academic Graph (MAG) is a comprehensive index of scientific publications in both journals and conferences(1). In November 2017, we downloaded 77,642,549 publications through the authorized API, freely provided by Microsoft Research available at https://www.microsoft.com/en-us/research/project/microsoft-academic-graph/. These publications were produced by 88,223,538 authors who contributed a total of 211,897,481 authorships.

C. DBLP. The DBLP Computer Science Bibliography contains 4,181,940 publications from computer science journals and conference proceedings (downloaded June 5th, 2018, https://dblp.uni-trier.de). We consider all articles, review articles, proceedings, book chapters, and dissertations published between 1970 and 2010, and exclude all other types of documents (e.g. webpages and notes), that are generally not peer-reviewed. These publications were produced by 2,129,492 authors who contributed a total of 12,090,783 authorships.

2. Data pre-processing

A. Identifying scientific careers. While the problem of name disambiguation for scientific publications is notoriously difficult, the scientific community has recognized several disambiguation procedures that effectively capture scientific careers. Here, to demonstrate the robustness of our results to database bias and author disambiguation errors, we replicated our analysis in three databases, each with its own strengths and weakness. All three of the data sets we used (WoS, MAG, and DBLP) maintain unique author identifiers based on a different name disambiguation procedure. The WoS and MAG use their own proprietary algorithms which have been successfully used to study scientific careers (for example, see WoS(2), and MAG(3)). While the specifics of the algorithms are not available, it is reasonable to assume that both algorithms are on par, if not far better than prevailing methods developed by independent academic groups. For instance, the MAG processes online CVs and Wikipedia profiles to associate individual authors with their papers. Additionally, both algorithms incorporate the self-curated career profiles provided by the Open Researcher and Contributor ID (ORCID). On the other hand, the DBLP name disambiguation is based on a unique identifier assigned to authors when manuscripts are submitted to registered Computer Science conferences or journals. Thus, the DBLP database has arguably the most reliable name disambiguation available in a bibliometric database(4), and has also been used in several peer-reviewed studies to study scientific careers(5, 6).

While many of the name disambiguation algorithms are able to reconstruct the careers for authors with European names, they often have difficulty disambiguating the careers of authors with Asian names. This, combined with the known issues inferring the gender of Asian names (see below), motivates us to adapt a conservative approach and exclude all researchers from China (mainland, Hong Kong, Macau, & Taiwan), the Democratic People's Republic of Korea, Japan, Malaysia, the Republic of Korea, and Singapore.

Critically, by replicating our study in three different databases, each with an independent method for name disambiguation, we argue that any possible errors resulting from misappropriated or missing publications are negligible.

B. Career selection criteria. In order to study comprehensive scientific careers, we limit our analysis to authors that: (i) have authored at least two papers, (ii) their publication careers span more than one year (365 days), (iii) have an average annual publication rate of less than 20 papers per year, (iv) have published their last article on or before Dec 31st, 2010. Our main conclusions do not change if more stringent selection criteria or modified filters are used to select the subset of scientists.

C. Country label. To facilitate the assignment of author gender (Section 2.E) and analyze national variations in the gender gap, we associate each author to a single country as follows. In the WoS, many authorships are indexed along with an affiliation address, including an institution name, street address, city, zipcode and country. For each author, we identify all authorships with a known affiliation address and keep only the country of the affiliation. We then assign a country label to an author based on the most frequently occurring country of affiliation. This frequency-based method results in a country label for a total of 1,876,950 authors.

We also considered an alternative method for country assignment in which the earliest country affiliation was used for each author. This second method disagrees with the frequency-based approach for only 58,576 (3.12%) of authors, and does not qualitatively affect results.

For the country-specific analysis, we disregard countries with less than 100 male or 100 female authors because the sample size is not sufficiently large to produce reliable statistics. This results in the following 83 countries reported in country-specific analysis in the main manuscript: Algeria, Argentina, Armenia, Australia, Austria, Bangladesh, Belarus, Belgium, Bolivia, Bulgaria, Cameroon, Canada, Chile, Colombia, Costa Rica, Croatia, Cuba, Cyprus, Czech Republic, Denmark, Ecuador, Egypt, Estonia, Finland, France, Gabon, Germany, Greece, Hungary, Iceland, India, Indonesia, Iran, Ireland, Israel, Italy, Jamaica, Jordan, Kazakhstan, Kenya, Kuwait, Latvia, Lebanon, Lithuania, Luxembourg, Macedonia, Madagascar, Mexico, Morocco, Netherlands, New Zealand, Nigeria, Norway, Pakistan, Peru, Philippines, Poland, Portugal, Qatar, Romania, Russia, Saudi Arabia, Senegal, Serbia, Slovakia, Slovenia, South Africa, Spain, Sri Lanka, Sweden, Switzerland, Tanzania, Thailand, Tunisia, Turkey, Uganda, Ukraine, United Arab Emirates, United Kingdom, United States, Uruguay, Uzbekistan, Venezuela.

D. Affiliation rank. It has been suggested that the author's primary affiliation contributes significantly towards the overall productivity(7). We collected the ranking information from The Times Higher Education World University Rankings 2019[*], a global ranking that indexes more than 1,250 universities. We then associate authors with universities by examining the affiliations in their publications. Considering university names could be spelled in multiple ways, such as abbreviations, we queried every affiliation name in the Web of Science publication data, as well as all university names in the Times Higher Education World University Rankings, with Google Maps to disambiguate those variations into unique university names. Each author is then assigned the rank of the highest ranked institute to which she or he is affiliated over the course of the career. Among 1,876,950 authors with at least one affiliation recorded, 1,296,995 authors have been aligned to an institute rank.

E. Gender assignment. In the absence of gender information for authors in the WoS, MAG, and DBLP we infer author gender based on author name and country. Specifically, we used a commercially available service *Genderize.io*[†] which integrates publicly available census statistics to build a name database mapping a first name to a binary gender label. When available, the accuracy of this procedure can be increased by specifying a country, although it is not required. This gender assignment strategy has also been successfully employed in several academic research projects(3, 5, 8). Due to a low accuracy of the gender assignment algorithm for East Asian names, when the country information was available (see section 2.C), we excluded all researchers from China (mainland, Hong Kong, Macau, & Taiwan), the Democratic People's Republic of Korea, Japan, Malaysia, the Republic of Korea, and Singapore. We also excluded researchers from Brazil due to poor performance in gender identification as reported in Kamiri et al (2016).

E.1. WoS and MAG authorship alignment. A practical challenge lies in the fact that the WoS dataset records the full first name of authors on most papers published after 2006, while the authorships are recorded with initials only for most papers before 2006. Among a total of 7,817,639 authors in the Web of Science dataset, only 2,171,290 of them have the full first name recorded for at least one authorship. Therefore, we leveraged our access to multiple datasets to help complete the missing metadata from the papers. Specifically, we aligned papers in the WoS to MAG based on the following criteria: (a) both papers are published in the same year, (b) both papers have identical sets of author last names, (c) the two papers differ in title by no more than 25%, estimated by the Levenshtein distance between two titles divided by the length of the WoS paper title. Such matches were found for 23,615,112 papers. We aligned authorships in each paper pair by comparing first initial and last name. For example, if a WoS paper records an author "J. Smith" and its matched paper in MAG records "John Smith", we complete the authorship "J Smith" with "John Smith". We skipped papers with multiple authors sharing the same last name. This procedure allowed us to complete the first name for additional 1,322,870 authors.

Note that this procedure only filled in missing metadata at the level of individual papers. The alignment between WoS and MAG was not used to infer features of an author's career.

E.2. Gender label inference. Out of the 3,427,232 WoS authors with full first name, we successfully inferred the gender of 3,003,815 authors, including 2,146,926 male authors and 856,889 female authors.

E.3. Gender label accuracy. As reported in Karimi et al. (2016) (9), genderize.io achieves a minimum accuracy of 82%, with an F1 score of 90% for females and 86% for males. To assess the accuracy of the gender assignment process for our data, we compared the inferred gender labels of authors in the WoS with a ground truth benchmark dataset consisting of 2,000 male and female full names manually collected in Lariviere et al. (2013) (10). Among the 1,512 author names that overlap with our dataset, 1,425 have inferred gender labels that agree with the ground truth, resulting in an accuracy of 94.25%.

E.4. WoS disambiguation gender invariance. To measure potential gender bias in author disambiguation, we used author careers curated by librarians from the American Mathematical Society and available on the MathSciNet, https://mathscinet.ams.org/mathscinet/index.html. The MathSciNet resource represents one of the only publicly available, large-scale databases with human curated publication profiles and sufficient historical coverage. MathSciNet indexes non-English language journals and peer-reviewed conference proceedings, so we expect its careers to cover many more publications than indexed from the WoS. However, we do not expect these indexing differences to introduce any gender bias, thus we feel it provides a definitive ground truth of author careers in mathematics.

Our experiment was conducted as follows. We selected 290 authors from the WoS who predominately published in mathematics, evenly distributed between male and female authors (145 male, 145 female). Of these 290 authors, we could

[*] https://www.timeshighereducation.com/world-university-rankings/2019/world-ranking#!/page/0/length/25/sort_by/rank/sort_order/asc/cols/stats, accessed May 2019

[†] https://genderize.io/

uniquely match 270 author profiles from the WoS to the MathSciNet using only the full author name, and an additional 8 male and 8 female authors using a combination of full name and article titles. We could not match 4 female author careers. This resulted in a total of 109 matched pairs (218 author careers) created such that the male and female authors have exactly the same number of publications in the WoS.

In this sample of 218 authors, we found that the MathSciNet contained an average of 11.6 more peer-reviewed publications for female authors, and an average of 13.9 more peer-reviewed publications for male authors. However, this difference is not statistically significant as rejected by a t-test with the test statistic of 0.89 and a pvalue of 0.38. Furthermore, we applied a Bayesian test for the difference in the means(11) to the number of publications, and found that 0 fell well within the 95% confidence interval for the difference in the means (mean of 0.46 inside of -2.2 to 2.75), allowing us to conclude that the means of these two distributions are statistical equivalent. Finally, we found that the difference in career lengths was 4.8 years and 6.6 years respectively, but this difference was not statistically significant as rejected by a t-test with the test statistic of 1.29 and a pvalue of 0.20. In summary, these experiments provide evidence that the WoS author disambiguation algorithm does not introduce significant gender differences due to algorithmic error.

Finally, we note that MathSciNet profiles list all pen-names under which an author has published, allowing us to test if indeed, female last name changes would introduce a significant bias. Of the 141 matched female authors, 5 significantly changed their last names (different name or hyphenated name), while 4 males significantly changed their last names. However, following this same logic, name disambiguation algorithms would also be sensitive to alternative spellings of the last name. For example, many Russian last names contain letters from the modern Russian alphabet that have multiple equivalents in the english alphabet. We found that 15 women had multiple last names, and 15 men had multiple last names. This suggests that multiple last names should not be expected to introduce a significant bias in our dataset.

F. Citation count and normalization.

F.1. Citations within Web of Science. We only count citations in which both the citing paper and cited paper appear within the WoS database.

F.2. Removing self-citations. It has previously been shown that male scientists are more likely to cite their own papers than female scientists(12). Therefore, in all measures of impact, we removed all self-citations based on the overlap between authorships in the citing paper and cited papers. We also replicated our analysis while keeping all self-citations and found no qualitative difference in our primary conclusions.

F.3. Citation normalization. Citation-based measures of impact are affected by two major problems: (1) citations follow different dynamics for different papers(13) and (2) the average number of citations changes over time(14). To overcome the first problem, we focused on the total number of citations each paper received within 10 years after its publication, c_{10}, as a measure of its scientific impact. We corrected for the second problem by normalizing the c_{10} for each paper by the average c_{10} of papers published in the same year, and multiplying by 12 (an arbitrary constant that does not quantitatively affect any of our analysis but restores the normalized citation count back to a realistic value). The resulting normalized c_{10} score thus provides a consistent measure of impact across decades.

G. Discipline hierarchy. We used a classification of scientific fields as defined in Wikipedia[‡] to re-organize 153 WoS categories into 75 disciplines. See S1 for the details of the mapping.

Each paper is assigned one or more disciplines among the 75 Wikipedia disciplines based on its original WoS category label(s). 3,117,710 (39.66%) authors have all papers assigned to a single discipline, while the remaining 4,742,941 (60.34%) authors are associated with at least two disciplines. For each author with multiple disciplines, we assign with a single discipline label as the most frequently occurring one. 3,728,442 (78.61%) of 4,742,941 authors with multiple disciplines have the most frequent discipline occurring in more than half of his/her papers.

While some disciplines were associated with many authors (e.g. Heath Sciences has 584,628 authors), many were only associated with a few authors. Therefore, we limit the majority of our analysis to the top 12 disciplines based on total population: **Health Science, Biology, Chemistry, Engineering,Physics, Computer Science, Psychology, Agronomy, Mathematics, Environmental science, Political Science, Applied physics**. These 12 disciplines cover 90.3% of the population. The remaining 9.7% of the population are grouped into the 13th category **Others** containing 4 fields in Formal Sciences (Decision theory, Logic, Statistics, Systems theory), 9 fields in Natural Sciences (Botany, Earth science, Ecology, Geology, Human biology, Meteorology, Oceanography, Space Science and Astronomy, Zoology), 14 fields in Applied Sciences (Applied chemistry, Applied linguistics, Applied mathematics, Architecture, Computing technology, Education, Electronics, Energy storage, Energy technology, Forensic science, Management, Microtechnology, Military science, Spatial science), 30 fields in Social Sciences (Anthropology, Business studies, Civics, Cognitive Science, Criminology, Cultural studies, Demography, Development studies, Economics, Education, Environmental studies, Gender and sexuality studies, Geography, Gerontology, Industrial relations, Information science, International studies, Law, Legal management, Library science, Linguistics, Management, Media studies, Paralegal studies, Planning, Public administration, Social work, Sociology, Sustainability studies, Sustainable development), 5 fields in Arts and Humanities (Arts, History, Languages and literature, Philosophy, Theology), and one last field "Unknown" that we failed to map to any Wikipedia discipline.

[‡] Last accessed August 2018. Branches of science (Wikipedia), Outline of natural science (Wikipedia), Outline of social science (Wikipedia), Outline of applied science (Wikipedia)

H. Data summary. After all data processing steps were completed, we consider 1,523,002 WoS authors (1,110,194 male, 412,808 female), contributing 18,561,863 authorships to 12,959,506 papers, across 13 disciplines and 83 countries. From this population, the country and affiliation information is available for only 103,104 authors (34,139 female and 68,965 male). This subset is used for the country specific statistics, and for a more constrained matching experiment.

3. Indicators

A. Characterizing the scientific career.

1. **Total productivity** of a scientist is defined as the total number of publications published by a specific author.

2. **Career Length** of a scientist is defined as the difference between the date of publication for their first and last publications. The career length is naturally found at the resolution of days, while in coarser scenarios we report career length in years by dividing by 365.

3. **Annual Productivity** of a scientist is calculated as the ratio of total productivity to career length, i.e., (the total number of papers) / (the days between the first and last publications / 365).

4. **Total impact** is defined as the sum of normalized c_{10} scores for each paper published by a specific author.

5. **Academic Age** of a scientist counts the number of years since his/her first publication. For example, a scientist whose first publication was in 1991, will have an academic age of 5 in 1995.

6. **Dropout** of a scientist occurs when the scientist publishes their final paper recorded in the data.

B. Characterizing the scientific population.

1. **Gender gap** is calculated for each indicator as the relative difference, i.e., the difference between the mean female and male values divided by the value of the male indicator.

2. **Dropout rate** of a group of scientists (e.g., those at the same age etc.) is the proportion of scientists who dropout from the group in the next year.

4. Methods

A. Statistical significance. For each measurement of scientific performance, we report the gender gap as the difference between the mean value for female and male scientists. Additionally, we compute the statistical significance of the gap using the unpaired two-tailed Welch's t-test to detect whether two samples with unequal size and unequal variance deviate from the null hypothesis that the two distributions (female and male) have the same mean. The corresponding p-values, indicating the statistical significance of the test, are reported in Tables S3, S4, S5, S6.

B. Career length matching. In order to assess the relationship between career length and total productivity, we conducted a matching experiment as follows. We first constructed a matched baseline population, in which, for each female author, we identified, without-replacement, a male author from the same discipline. If multiple male authors were found, we randomly selected one to match without replacement. This process consistently produced 412,797 matched pairs. To account for the inherent randomness in this procedure, the experiment was replicated 50 times, and the reported performance was averaged over all random trials. The standard deviation over the trials is near zero for both the productivity and impact gaps. To provide an accurate baseline for comparison, we recalculated the gender gaps in productivity and impact (shown in the main text Figure 3D,E, middle bars). The gender gaps in the discipline matched population differ slightly from those observed in the total population. We then created our second experimental population, as a subset of the first, in which we matched each female author to a male author from the same discipline and with exactly the same career length.

Several studies have suggested that the affiliation of authors might be an important factor influencing their productivity. Since affiliation information is less common in the WoS, we explore its possible role as a confounding variable in a second matching experiment. Recall that we have country and affiliation information for only 103,104 authors (34,139 female and 68,965 male). We then assigned each author to a group based on their highest ranking affiliation, for which we binned the institutions by rank into 15 equal volume bins; no significant difference occurs for other choices of the affiliation binning. The matched baseline population, in which, for each female author, we identified, without-replacement, a male author from the same country, discipline, and with the same affiliation rank bin consistently produced 32,782 matched pairs. The gender gaps in productivity and impact are significantly larger in the matched populations (Fig. S1**A,B**), likely due to the fact that the coverage in country and affiliation information is biased towards more recent and senior scientists. We then created our second experimental population, as a subset of the first, in which we matched each female author to a male author from the same country, discipline, with the same affiliation rank bin, and with exactly the same career length. This process consistently produced 25,033 matched pairs. Once again, the additional constraint based on career length significantly reduces both the productivity and impact gender gaps.

C. Annual productivity matching. We also conducted a similar experiment controlling for the annual productivity. Specifically, we constructed another set of matched samples in which we identified for each female, a male author from the same country and discipline, with a nearly identical annual productivity based on grouping authors into bins by annual productivity: [0.1 papers/year, 0.2 papers/year), [0.2 papers/year, 0.3 papers/year), etc. The approximation occurs because annual productivity is a real-valued number. As seen in Fig. S3**A,B**, controlling for annual productivity actually increases gender gaps in both the total productivity and total impact, although the increase is small (1.6% and 0% respectively). The lack of a significant change in the total productivity gender gap further emphasizes the importance of career length as the dominating factor.

D. Total productivity matching. Our third matching experiment controlled for the total productivity and explored the resulting change in impact. Specifically, we constructed another set of matched samples in which we identified for each female author, a male author from the same country, discipline, and approximately the same affiliation rank. In this population, the gender gap in career impact was 50.7% in favor of male authors. We then created our second experimental population, as a subset of the first, in which we matched each female author to a male author from the same country, discipline, with approximately the same affiliation rank, and with exactly the same total productivity. With the addition of matching on total productivity, the impact gap actually flips in favor of female scientists who receives an average of 1.9% more citations. We report the mean impact gap over 100 randomized trials and the standard deviation for the impact gap is nearly zero.

E. Relationship between productivity and number of collaborators. The gender gap in total productivity has an important implication for any reported gender gaps in collaboration and the subsequent structure of collaboration networks. Here, we test for this relationship by using a matching experiment in which we selected a male author from the same country, discipline, and affiliation rank. We then calculate the total number of collaborators that co-authored at least one publication, and find a substantial gender gap (Fig. S2, left): while men collaborate with an average of 36.6 co-authors, female authors collaborate with an average of 23.5 co-authors, a gender gap of 35.8%. Next, a subset of this matched population was chosen such that the male and female authors published exactly the same number of articles throughout their careers (Fig. S2, right). We see that in this final matched population, the gender gap in number of collaborators actually switches to 4.1% in favor of female authors.

F. Controlling for the dropout rate. We introduce an experiment that simulates an alternative scientific population in which we manipulate the dropout rate of scientists. While it would be difficult to retroactively identify the potential publications a scientist would have published if their career did not terminate in a given year, we can more easily randomly terminate the careers of scientists earlier than reality. Here, we use this technique to eliminate the gender gap in dropout rate, and test for the effects on the productivity and impact gender gaps.

As shown in the main text, Fig. 4**A**, the age-dependent dropout rate for women is always higher than the male dropout rate. To correct for this gender gap, we raise the dropout rate for male scientists to match that of the female scientists. Specifically, for a given year, we find the difference between the male and female dropout rates, and identify how many more men would need to dropout in order to equalize the rate. We then randomly select male scientists who otherwise would not have left the population the following year (we do not consider the remainder of the career length when selecting scientists) and terminate their careers. A selected male scientist keeps all publications until this age, while his authorships on all later publications are discarded (only the authorships are removed from the data, the career termination of a selected scientist does not affect his collaborators or citations). To account for the inherent randomness in this procedure, the experiment has been replicated 100 times and we report the mean gender gaps, while the standard deviation is near zero.

G. Career pauses. Previous research has suggested that the time between publications could be an important factor in understanding gender differences in the productivity of male and female authors. To explore this relationship, we first looked at the longest pause between publications (in number of days) for each author in our dataset. As shown in Fig. S11**A**, while there is a small difference in the distributions of longest pause for male and female authors, this difference actually suggests males have longer pauses during their careers. Indeed, on average, the longest pause in a male publication career is 1583 days, while the longest pause in a female publication career is only 1411 days (due to the large sample sizes, this difference is statistically significant as verified by a Welch test, with a test statistic of 60.84 and a p-value < 10-10).

It is also interesting to note that the length of the longest pause in between publications is highly correlated with the total career length (Spearman correlation of 0.75). However, even if we control for career length, we continue to find that male careers have slightly longer pauses compared to female careers (Fig. S11**B**) for careers less than 24 years (covering 87.87% of male authors, and 93.14% of female authors) while female authors have slightly longer pauses for careers longer than 24 years (covering 12.13% of male authors, and 6.86% of female authors). Since we observed significant differences in the dropout rate of female and male authors throughout all stages of their careers (see main text Figure 4A), we do not believe the difference in career pause length is a primary factor driving the gender differences in productivity, impact, and career length. However, future research could explore if career pauses can be differentiated from career termination events, providing a potential avenue for retention of female scientists in the academic workforce.

We also conducted a second experiment in which we removed all years in which an author had 0 publications and then reproduced our key observations of gender differences originally reported in the main text, Figure 2. As shown in Fig. S12**P**, an average male author will publish for 6.17 active years while a female author will publish for 5.22 active years, resulting in a gender gap of 15% more active years for male authors. This is very similar to our originally reported gender gap of 16% longer careers for male authors. Using only active years in the calculation for annual productivity reveals that male authors publish

an average of 1.58 articles per active years, and female authors publish an average of 1.53 articles per active year, resulting in a gender gap of 3%. While statistically significant, this gap is considerably smaller than the 27% gap in productivity.

In conclusion, pauses in academic publishing don't strongly effect on the gender differences reported here. However, this analysis captures only two aspects of publishing pauses, and does not rule out the importance of publication pauses for the success of academic careers. For example, it has been demonstrated that annual publication rates vary significantly over the course of an academic career, and do not all follow canonical trajectories(6). This suggests that additional factors beyond the length of the publication pause, such as the timing of that pause relative to the rest of the career, could be associated with significant gender differences. The methodology we introduce here may allow for further exploration of the effect of pauses in academic publishing on academic careers.

5. Detailed results on Web of Science

A. Distributions of measurements. Fig. S5**A-D** reports the rank distributions of the four major indicators for male and female scientists. For each indicator type, we rank scientists from highest to lowest (denoted as the percentile of scientists with higher performance), and report the performance against percentiles. The difference between the rank distributions shows that, on average, male scientists have more publications and citations, and have longer careers compared to the female scientists. The gender inequality is most significant among top scientists (insets in all four panels). In contrast, male and female scientists look very similar when measured by annual productivity and citation rate.

B. Statistics and gender gaps in each discipline, country, and year. The gender gaps in scientific measurements across all countries (Fig. 2**B,G,L,Q** from the main text) is reproduced and fully labeled in Fig. S6**A-D**. Tables S3 and S4 report the statistics of male and female scientists broken down by discipline and country. Each row reports the population size and mean performance indicators of male (in blue) and female (in orange) authors. The standard error is reported as one standard deviation. Table S5 and S6 report the statistics of male and female scientists grouped by the year they start and finish their scientific careers, respectively.

The detailed relationship between the gender gap in career length and total productivity across all countries is shown in Fig. S7 as a fully labeled version of Fig. 3**B** from the main text.

6. Replication in other databases

A. Microsoft Academic Graph. Following the procedure for the Web of Science (Section 1), we identified the genders of 5,856,109 male and 2,622,594 female authors who published a total of 77,642,549 articles in the MAG. Fig. S8**A-C** shows the gender gaps in total productivity, annual productivity and career length in the MAG. Similar to the findings reported for the WoS in the main text, we find large gender gaps in total productivity and career length, while male and female scientists differ only slightly in annual productivity. Likewise, we find that female scientists consistently have a higher dropout rate than male scientists (Fig. S9**A**) which results in a separation of the survival curves (Fig. S9**B**).

B. DBLP. To prepare the DBLP data, we followed the procedure for the Web of Science (Section 1), with the following modification. Because affiliation information for the DBLP is largely absent, we could not leverage location information to assist in the gender assignment. Instead, we compiled a list of 107,675 unique Chinese first names from the Chinese Biographical Database Project (https://projects.iq.harvard.edu/cbdb/home) and 564 unique Korean first names from wikipedia (https://en.wikipedia.org/wiki/List_of_Korean_given_names) and removed any author with a matching name from the dataset. After cleaning, we identified the genders of 301,150 male and 69,473 female authors who published a total of 1,740,482 articles in the DBLP.

References

1. Sinha A, et al. (2015) An overview of microsoft academic service (MAS) and applications. *Proceedings of the 24th International Conference on World Wide Web* pp. 243–246.
2. Liu L, et al. (2018) Hot streaks in artistic, cultural, and scientific careers. *Nature* 559(7714):396–399.
3. AlShebli BK, Rahwan T, Woon WL (2018) The preeminence of ethnic diversity in scientific collaboration. *Nature Communications* 9(1):5163.
4. Reuther P, Walter B, Ley M, Weber A, Klink S (2006) *Managing the Quality of Person Names in DBLP*, Lecture Notes in Computer Science. (Springer, Berlin, Heidelberg), p. 508–511.
5. Jadidi M, Karimi F, Lietz H, Wagner C (2017) Gender disparities in science? dropout, productivity, collaborations and success of male and female computer scientists. *Advances in Complex Systems* p. 1750011.
6. Way SF, Morgan AC, Clauset A, Larremore DB (2017) The misleading narrative of the canonical faculty productivity trajectory. *Proceedings of the National Academy of Sciences* 114(44):E9216–E9223.
7. Way SF, Morgan AC, Larremore DB, Clauset A (2019) Productivity, prominence, and the effects of academic environment. *Proceedings of the National Academy of Sciences* p. 201817431.
8. Holman L, Stuart-Fox D, Hauser CE (2018) The gender gap in science: How long until women are equally represented? *PLOS Biology* 16(4):e2004956.

9. Karimi F, Wagner C, Lemmerich F, Jadidi M, Strohmaier M (2016) Inferring gender from names on the web: A comparative evaluation of gender detection methods in *Proceedings of the 25th International Conference Companion on World Wide Web*, WWW '16 Companion. (International World Wide Web Conferences Steering Committee), pp. 53–54.
10. Lariviere V, Ni C, Gingras Y, Cronin B, Sugimoto CR (2013) Global gender disparities in science. *Nature* 504(7479):211–213.
11. Kruschke JK (2013) Bayesian estimation supersedes the t test. *Journal of Experimental Psychology: General* 142(2):573–603.
12. King MM, Bergstrom CT, Correll SJ, Jacquet J, West JD (2017) Men set their own cites high: Gender and self-citation across fields and over time. *Socius* 3:2378023117738903.
13. Wang D, Song C, Barabási AL (2013) Quantifying long-term scientific impact. *Science* 342(6154):127–132.
14. Sinatra R, Wang D, Deville P, Song C, Barabàsi AL (2016) Quantifying the evolution of individual scientific impact. *Science* 354(6312):aaf5239.

7. Tables and Figures

Web of Science category	Re-organized field
Mathematics	a.c Mathematics
Computer Science	a.f Theoretical computer science
Physics, Thermodynamics, Mechanics, Acoustics, Crystallography	b.a Physical science - Physics
Chemistry, Electrochemistry, Geochemistry & Geophysics, Spectroscopy	b.b Physical science - Chemistry
Oceanography	b.e Physical science - Oceanography
Geology	b.f Physical science - Geology
Meteorology & Atmospheric Sciences	b.g Physical science - Meteorology
Astronomy & Astrophysics	b.h Physical science - Space Science or Astronomy
Biochemistry & Molecular Biology, Cell Biology, Plant Sciences, Microbiology, Developmental Biology, Evolutionary Biology, Biophysics, Mathematical & Computational Biology, Genetics & Heredity, Reproductive Biology, Paleontology, Parasitology, Virology, Mycology	b.i Life science - Biology
Zoology, Entomology	b.j Life science - Zoology
Agriculture, Food Science & Technology, Forestry, Transplantation	c.a Agronomy
Architecture, Construction & Building Technology	c.b. Architecture
Education & Educational Research	c.e Education
Energy & Fuels	c.g Energy technology
Materials Science, Engineering, Polymer Science, Automation & Control Systems, Mining & Mineral Processing, Mineralogy, Marine & Freshwater Biology, Robotics, Metallurgy & Metallurgical Engineering, Biotechnology & Applied Microbiology, Instruments & Instrumentation, Telecommunications	c.i Engineering
Environmental Sciences & Ecology, Fisheries	c.j Environmental science

General & Internal Medicine, Health Care Sciences & Services, Integrative & Complementary Medicine, Legal Medicine, Radiology, Nuclear Medicine & Medical Imaging, Research & Experimental Medicine, Tropical Medicine, Critical Care Medicine, Dentistry, Oral Surgery & Medicine, Emergency Medicine, Toxicology, Surgery, Psychiatry, Physiology, Pharmacology & Pharmacy, Pediatrics, Pathology, Ophthalmology, Obstetrics & Gynecology, Nutrition & Dietetics, Nursing, Neurosciences & Neurology, Immunology, Infectious Diseases, Gastroenterology & Hepatology, Endocrinology & Metabolism, Dermatology, Cardiovascular System & Cardiology, Biodiversity & Conservation, Anatomy & Morphology, Urology & Nephrology, Veterinary Sciences, Oncology, Respiratory System, Hematology, Substance Abuse, Rheumatology, Otorhinolaryngology, Orthopedics, Anesthesiology, Allergy, Audiology & Speech-Language Pathology, Medical Informatics, Medical Laboratory Technology, Sport Sciences	c.l Health science
Operations Research & Management Science	c.n Management
Mathematical Methods In Social Sciences	c.o Applied mathematics
Nuclear Science & Technology, Optics	c.r Applied physics
Remote Sensing	c.s Spatial science
Anthropology, Archaeology, Religion, Ethnic Studies	d.a Anthropology
International Relations, Government & Law, Public, Environmental & Occupational Health	d.ab Political science
Psychology, Behavioral Sciences	d.ac Psychology
Public Administration	d.ad Public administration
Social Work	d.ae Social work
Sociology, Urban Studies, Social Issues	d.af Sociology
Business & Economics	d.b Business studies
Criminology & Penology	d.e Criminology
Cultural Studies, Asian Studies	d.f Cultural studies
Demography	d.g Demography
Women's Studies	d.l Gender and sexuality studies
Geography, Physical Geography, Area Studies	d.m Geography
Geriatrics & Gerontology	d.n Gerontology
Information Science & Library Science	d.q Information science
Linguistics	d.w Linguistics
Communication, Film, Radio & Television	d.y Media studies
Arts & Humanities - Other Topics, Life Sciences & Biomedicine - Other Topics, Rehabilitation, Physical Sciences - Other Topics, Water Resources, Technology - Other Topics, Imaging Science & Photographic Technology, Microscopy, Transportation, Social Sciences - Other Topics, Biomedical Social Sciences, Family Studies	e.a Unfiled
Art, Dance, Music, Theater	f.a Arts
Classics, History	f.b History
Literature	f.c Languages and literature
Philosophy, History & Philosophy of Science, Medical Ethics	f.d Philosophy

Table S1. The discipline hierarchy

Indicator	Female mean	Male mean	Gender gap	t-test statistic	t-test p-value
Total productivity	9.56±0.03	13.16±0.03	-27.38%	96.20	<1E-100
Total impact	175.49±0.86	252.35±0.87	-30.46%	62.05	<1E-100
Career length	9.26±0.01	11.02±0.01	-15.91%	109.07	<1E-100
Annual productivity	1.32±0.00	1.33±0.00	-0.88%	6.24	4.39E-10

Table S2. Academic performance. In each row we report the average measurements (± 1 standard error) of all female (orange) and male (blue) scientists for the total productivity, the total impact, career length and annual productivity. We also supply the test statistics for the difference of means between male and female scientists using the two-tailed Welch's t-test.

Discipline	Population	Total productivity	Total impact	Career length	Annual productivity
Agronomy	26,550	13.16±0.13	148.94±2.54	12.13±0.06	1.22±0.01
	9,403	9.01±0.16	97.30±2.03	9.63±0.08	1.24±0.01
		-31.5% (3E-88)	-34.7% (3E-58)	-20.7% (3E-122)	2.1% (2E-02)
Applied physics	15,662	8.74±0.12	90.57±1.99	9.05±0.06	1.31±0.01
	2,700	8.06±0.23	75.85±5.49	8.81±0.14	1.28±0.02
		-7.8% (9E-03)	-16.3% (6E-03)	-2.6% (2E-01)	-1.9% (2E-01)
Biology	107,219	16.56±0.08	435.97±3.40	12.31±0.03	1.38±0.00
	64,108	10.31±0.07	261.61±2.45	9.90±0.04	1.28±0.00
		-37.7% (0E+00)	-40.0% (0E+00)	-19.6% (0E+00)	-7.5% (6E-113)
Chemistry	114,381	16.07±0.09	269.91±1.89	11.89±0.03	1.45±0.00
	35,553	10.44±0.09	147.99±2.18	9.61±0.05	1.40±0.01
		-35.1% (0E+00)	-45.2% (0E+00)	-19.2% (0E+00)	-2.9% (1E-11)
Computer science	29,557	5.36±0.03	49.00±0.91	7.04±0.03	1.15±0.00
	5,660	4.95±0.06	35.93±1.17	6.45±0.07	1.21±0.01
		-7.8% (7E-09)	-26.7% (2E-19)	-8.4% (7E-15)	5.2% (2E-06)
Engineering	122,841	8.19±0.05	90.74±0.89	9.01±0.02	1.20±0.00
	26,396	7.12±0.08	79.12±1.10	8.24±0.04	1.23±0.01
		-13.0% (5E-34)	-12.8% (4E-14)	-8.5% (4E-51)	2.6% (4E-07)
Environment	18,271	9.01±0.12	152.64±2.88	11.02±0.07	1.05±0.01
	5,950	7.22±0.13	126.58±2.63	9.14±0.09	1.09±0.01
		-19.9% (4E-31)	-17.1% (8E-11)	-17.0% (1E-60)	4.5% (4E-05)
Health science	391,372	16.08±0.05	306.68±1.23	11.22±0.02	1.51±0.00
	175,174	10.95±0.05	205.45±1.24	9.21±0.02	1.46±0.00
		-31.9% (0E+00)	-33.0% (0E+00)	-17.9% (0E+00)	-3.0% (3E-41)
Mathematics	28,761	7.13±0.06	59.67±1.10	10.85±0.06	0.95±0.00
	5,154	5.55±0.10	36.73±1.83	9.07±0.11	0.94±0.01
		-22.1% (9E-41)	-38.4% (9E-25)	-16.4% (3E-46)	-0.8% (4E-01)
Others	135,270	7.57±0.04	127.74±1.21	10.28±0.03	1.01±0.00
	44,731	5.96±0.05	102.50±1.69	8.77±0.04	1.02±0.00
		-21.3% (1E-149)	-19.8% (2E-30)	-14.7% (2E-238)	0.9% (3E-02)
Physics	67,772	16.98±0.12	304.81±3.12	12.19±0.04	1.53±0.00
	12,292	13.66±0.20	205.63±5.31	10.83±0.09	1.57±0.01
		-19.5% (5E-40)	-32.5% (4E-55)	-11.1% (4E-42)	2.1% (1E-02)
Political science	15,896	7.46±0.11	128.16±3.22	10.39±0.08	1.00±0.01
	7,320	7.13±0.13	132.37±4.19	8.91±0.09	1.10±0.01
		-4.4% (6E-02)	3.3% (4E-01)	-14.3% (2E-38)	9.4% (4E-16)
Psychology	36,619	7.43±0.06	123.56±2.01	9.67±0.04	1.07±0.00
	18,356	5.69±0.05	95.32±1.58	8.35±0.06	1.03±0.01
		-23.5% (5E-78)	-22.9% (1E-27)	-13.7% (1E-73)	-4.6% (4E-13)

Table S3. Academic performance in disciplines. In each cell we report the average measurements of male (blue) and female (orange) scientists, with standard errors. A third row reports the gender gap in percentage and p-value in parentheses. The p-value is calculated with two-tailed Welch's t-test to detect whether two samples with unequal size and unequal variance have identical mean.

Country	Population	Total productivity	Total impact	Career length	Annual productivity
Algeria	134<br>42	7.16±0.62<br>6.76±0.68<br>-5.5% (7E-01)	64.77±9.79<br>48.72±8.96<br>-24.8% (3E-01)	12.96±0.69<br>10.71±0.74<br>-17.3% (3E-02)	0.73±0.04<br>0.80±0.07<br>8.8% (4E-01)
Argentina	1,025<br>961	13.57±0.59<br>10.25±0.34<br>-24.4% (2E-06)	125.17±8.94<br>85.19±4.98<br>-31.9% (8E-05)	15.78±0.33<br>13.52±0.25<br>-14.3% (4E-08)	0.91±0.02<br>0.88±0.02<br>-2.5% (5E-01)
Armenia	38<br>23	14.84±2.50<br>6.30±0.80<br>-57.5% (6E-03)	89.84±35.70<br>28.71±10.77<br>-68.0% (1E-01)	15.79±1.74<br>13.39±1.89<br>-15.2% (4E-01)	1.13±0.20<br>0.63±0.06<br>-44.4% (6E-02)
Australia	4,773<br>2,843	18.38±0.44<br>11.51±0.31<br>-37.4% (2E-35)	350.36±10.69<br>234.67±9.14<br>-33.0% (3E-14)	14.82±0.15<br>11.33±0.14<br>-23.6% (4E-60)	1.26±0.02<br>1.19±0.02<br>-5.7% (2E-03)
Austria	1,783<br>805	22.52±0.80<br>12.90±0.44<br>-42.7% (7E-21)	280.45±11.67<br>175.45±8.46<br>-37.4% (2E-12)	13.76±0.22<br>9.93±0.20<br>-27.8% (2E-32)	1.61±0.04<br>1.57±0.04<br>-2.8% (4E-01)
Bangladesh	97<br>34	10.37±1.31<br>8.38±1.51<br>-19.2% (3E-01)	77.61±12.43<br>86.04±39.43<br>10.9% (8E-01)	14.58±0.97<br>14.35±1.52<br>-1.5% (9E-01)	0.86±0.07<br>0.72±0.07<br>-16.0% (2E-01)
Belarus	83<br>93	16.89±2.47<br>10.02±0.90<br>-40.7% (6E-03)	61.99±10.55<br>56.50±16.54<br>-8.9% (8E-01)	17.00±1.17<br>15.54±0.98<br>-8.6% (3E-01)	1.07±0.06<br>0.82±0.06<br>-22.6% (1E-02)
Belgium	2,305<br>1,198	22.87±0.85<br>14.49±0.78<br>-36.6% (1E-14)	372.40±14.50<br>276.17±16.36<br>-25.8% (6E-05)	13.93±0.22<br>10.72±0.21<br>-23.1% (2E-25)	1.61±0.03<br>1.51±0.04<br>-6.3% (3E-02)
Bolivia	24<br>11	6.29±0.84<br>4.55±0.61<br>-27.8% (1E-01)	68.95±14.02<br>38.89±7.10<br>-43.6% (6E-02)	13.38±1.62<br>9.36±1.80<br>-30.0% (1E-01)	0.62±0.08<br>0.77±0.13<br>25.9% (3E-01)
Bulgaria	256<br>265	14.78±1.42<br>11.64±0.83<br>-21.2% (7E-02)	100.30±16.28<br>75.26±8.74<br>-25.0% (1E-01)	16.24±0.60<br>14.82±0.51<br>-8.8% (9E-02)	0.98±0.05<br>0.90±0.04<br>-7.9% (2E-01)
Cameroon	71<br>23	7.99±0.78<br>12.04±3.81<br>50.8% (3E-01)	69.10±11.40<br>240.35±103.98<br>247.8% (1E-01)	11.31±0.79<br>10.96±1.40<br>-3.1% (8E-01)	0.95±0.07<br>1.11±0.16<br>17.6% (3E-01)
Canada	7,840<br>4,450	18.46±0.38<br>11.52±0.26<br>-37.6% (3E-48)	353.74±10.11<br>232.59±6.73<br>-34.2% (5E-23)	14.44±0.13<br>11.17±0.11<br>-22.6% (2E-86)	1.27±0.01<br>1.17±0.01<br>-7.3% (3E-07)
Chile	678<br>324	11.58±0.72<br>10.10±0.63<br>-12.8% (1E-01)	120.24±12.57<br>89.70±9.26<br>-25.4% (4E-02)	14.16±0.37<br>13.20±0.50<br>-6.8% (1E-01)	0.93±0.02<br>0.89±0.04<br>-4.4% (3E-01)
Colombia	206<br>99	7.63±0.49<br>7.98±0.59<br>4.6% (7E-01)	90.55±11.04<br>94.87±16.40<br>4.8% (8E-01)	10.82±0.44<br>11.71±0.61<br>8.2% (3E-01)	0.95±0.05<br>0.87±0.06<br>-7.5% (4E-01)
Costa Rica	55<br>25	8.04±0.73<br>10.96±1.30<br>36.4% (7E-02)	58.53±7.75<br>125.97±37.48<br>115.2% (9E-02)	12.98±0.99<br>15.16±2.07<br>16.8% (3E-01)	0.85±0.08<br>0.87±0.08<br>2.3% (9E-01)
Croatia	462<br>370	13.50±0.84<br>11.36±0.64<br>-15.9% (4E-02)	83.16±7.18<br>70.29±8.14<br>-15.5% (2E-01)	14.73±0.38<br>13.29±0.45<br>-9.8% (2E-02)	0.95±0.03<br>1.04±0.04<br>9.7% (6E-02)
Cuba	125<br>138	10.86±0.99<br>11.04±1.64<br>1.6% (9E-01)	82.83±11.70<br>66.40±8.91<br>-19.8% (3E-01)	12.70±0.71<br>12.03±0.65<br>-5.3% (5E-01)	0.99±0.08<br>0.95±0.06<br>-4.2% (7E-01)
Cyprus	30<br>15	8.33±1.00<br>7.27±0.98<br>-12.8% (5E-01)	90.21±24.71<br>50.74±13.51<br>-43.8% (2E-01)	10.33±0.97<br>8.07±0.66<br>-21.9% (6E-02)	1.19±0.25<br>1.13±0.18<br>-5.4% (8E-01)
Czech Republic	1,116<br>557	19.72±0.97<br>11.38±0.51<br>-42.3% (8E-14)	171.97±11.81<br>98.70±6.93<br>-42.6% (8E-07)	16.14±0.35<br>11.42±0.36<br>-29.2% (3E-19)	1.23±0.03<br>1.30±0.04<br>5.5% (2E-01)
Denmark	1,612<br>759	19.34±0.70<br>12.51±0.63	416.23±20.30<br>266.88±21.86	14.51±0.26<br>11.28±0.29	1.37±0.03<br>1.28±0.03

		-35.3% (2E-11)	-35.9% (5E-07)	-22.2% (5E-17)	-6.8% (3E-02)
Ecuador	22<br>14	6.41±0.87<br>8.86±1.63<br>38.2% (3E-01)	104.76±27.14<br>98.31±20.42<br>-6.2% (9E-01)	10.50±0.98<br>14.00±3.20<br>33.3% (3E-01)	0.73±0.09<br>0.75±0.08<br>1.8% (9E-01)
Egypt	563<br>232	11.55±0.56<br>9.20±0.53<br>-20.3% (2E-03)	82.42±4.85<br>66.65±5.58<br>-19.1% (3E-02)	14.50±0.40<br>14.37±0.57<br>-0.9% (9E-01)	0.93±0.03<br>0.79±0.04<br>-14.9% (4E-03)
Estonia	122<br>86	12.19±1.26<br>10.27±1.34<br>-15.8% (3E-01)	142.46±25.31<br>131.38±38.34<br>-7.8% (8E-01)	13.48±0.71<br>12.12±0.88<br>-10.1% (2E-01)	1.00±0.06<br>0.98±0.06<br>-1.9% (8E-01)
Finland	1,573<br>1,117	19.68±0.86<br>13.72±0.55<br>-30.3% (8E-09)	313.46±17.70<br>253.39±15.87<br>-19.2% (1E-02)	14.20±0.23<br>11.40±0.22<br>-19.8% (2E-19)	1.32±0.03<br>1.30±0.02<br>-1.8% (5E-01)
France	10,708<br>6,487	26.13±0.40<br>16.71±0.29<br>-36.0% (2E-69)	398.36±7.59<br>283.79±7.63<br>-28.8% (5E-26)	16.41±0.11<br>13.48±0.12<br>-17.8% (1E-73)	1.41±0.01<br>1.28±0.01<br>-9.3% (8E-15)
Gabon	13<br>10	12.62±3.02<br>8.50±1.34<br>-32.6% (2E-01)	229.60±50.59<br>100.10±30.23<br>-56.4% (5E-02)	11.46±2.10<br>8.40±0.95<br>-26.7% (3E-01)	1.36±0.18<br>1.26±0.19<br>-7.3% (7E-01)
Germany	14,994<br>5,739	22.28±0.33<br>12.17±0.22<br>-45.4% (5E-139)	350.28±6.63<br>211.65±6.00<br>-39.6% (1E-58)	13.57±0.09<br>9.93±0.09<br>-26.8% (6E-198)	1.58±0.01<br>1.45±0.02<br>-8.4% (9E-13)
Greece	1,848<br>869	15.15±0.46<br>11.14±0.32<br>-26.5% (4E-12)	136.97±6.03<br>106.36±5.68<br>-22.3% (3E-04)	12.50±0.19<br>10.71±0.21<br>-14.3% (3E-10)	1.40±0.03<br>1.35±0.04<br>-3.5% (3E-01)
Hungary	1,083<br>567	18.67±0.95<br>13.16±0.82<br>-29.5% (8E-07)	176.87±13.59<br>126.29±9.98<br>-28.6% (3E-03)	16.05±0.36<br>13.32±0.39<br>-17.0% (4E-07)	1.19±0.03<br>1.24±0.04<br>4.3% (3E-01)
Iceland	91<br>40	11.79±1.14<br>10.97±1.22<br>-6.9% (7E-01)	379.82±65.52<br>595.79±156.63<br>56.9% (2E-01)	13.73±0.91<br>11.03±0.91<br>-19.7% (2E-02)	0.94±0.07<br>1.22±0.10<br>29.5% (3E-02)
India	3,537<br>1,789	14.46±0.42<br>11.46±0.40<br>-20.7% (1E-07)	126.90±4.66<br>104.07±6.31<br>-18.0% (5E-03)	14.51±0.17<br>14.02±0.24<br>-3.4% (1E-01)	1.20±0.02<br>1.07±0.02<br>-11.0% (4E-06)
Indonesia	86<br>51	8.35±0.77<br>9.43±1.27<br>13.0% (5E-01)	131.26±29.23<br>91.50±12.20<br>-30.3% (2E-01)	12.05±0.80<br>10.53±0.66<br>-12.6% (1E-01)	0.85±0.06<br>1.05±0.09<br>24.3% (1E-01)
Iran	701<br>176	9.12±0.46<br>8.09±0.45<br>-11.4% (1E-01)	81.58±8.24<br>83.30±15.39<br>2.1% (9E-01)	8.79±0.22<br>8.06±0.38<br>-8.3% (8E-02)	1.38±0.04<br>1.40±0.08<br>1.8% (8E-01)
Ireland	834<br>426	18.44±1.16<br>10.69±0.59<br>-42.0% (1E-09)	331.35±27.74<br>167.55±10.41<br>-49.4% (7E-08)	13.27±0.35<br>10.32±0.34<br>-22.3% (3E-09)	1.41±0.05<br>1.33±0.05<br>-5.7% (2E-01)
Israel	1,991<br>1,322	22.78±0.80<br>13.40±0.58<br>-41.2% (6E-19)	334.29±15.04<br>232.77±15.07<br>-30.4% (2E-05)	16.00±0.24<br>12.67±0.25<br>-20.8% (1E-20)	1.33±0.03<br>1.17±0.03<br>-12.5% (5E-06)
Italy	8,808<br>6,352	22.09±0.38<br>14.53±0.20<br>-34.2% (2E-70)	291.71±6.75<br>191.98±4.09<br>-34.2% (9E-36)	16.15±0.12<br>12.23±0.09<br>-24.3% (1E-137)	1.40±0.01<br>1.44±0.01<br>2.7% (5E-02)
Jamaica	38<br>20	14.63±3.66<br>14.10±3.38<br>-3.6% (9E-01)	114.76±51.74<br>132.10±46.41<br>15.1% (8E-01)	16.66±1.37<br>15.20±1.58<br>-8.8% (5E-01)	0.92±0.13<br>1.02±0.18<br>10.6% (6E-01)
Jordan	164<br>27	10.45±0.78<br>9.22±1.56<br>-11.8% (5E-01)	100.56±15.61<br>68.01±15.09<br>-32.4% (1E-01)	11.40±0.52<br>10.70±1.04<br>-6.1% (6E-01)	1.13±0.06<br>0.98±0.12<br>-13.4% (3E-01)
Kazakhstan	14<br>21	18.86±4.94<br>10.14±1.91<br>-46.2% (2E-01)	84.04±24.69<br>37.76±10.36<br>-55.1% (7E-02)	14.79±2.84<br>16.05±2.21<br>8.5% (7E-01)	1.45±0.19<br>0.85±0.14<br>-41.7% (5E-02)
Kenya	125<br>36	12.11±1.60<br>9.42±2.33	210.45±55.78<br>145.50±32.62	14.03±0.73<br>10.31±1.24	1.04±0.13<br>1.27±0.22

		-22.3% (4E-01)	-30.9% (3E-01)	-26.6% (2E-02)	22.7% (4E-01)
Kuwait	139<br>39	13.98±1.24<br>8.90±0.99<br>-36.3% (4E-03)	105.97±13.48<br>74.07±13.45<br>-30.1% (9E-02)	13.06±0.77<br>12.51±1.10<br>-4.2% (7E-01)	1.14±0.07<br>0.94±0.10<br>-17.9% (8E-02)
Latvia	36<br>46	12.53±1.55<br>10.35±1.40<br>-17.4% (3E-01)	86.58±16.07<br>45.23±6.44<br>-47.8% (3E-02)	13.97±1.23<br>12.57±1.04<br>-10.1% (4E-01)	1.04±0.09<br>1.04±0.10<br>-0.2% (1E+00)
Lebanon	121<br>61	11.97±0.88<br>9.57±1.28<br>-20.0% (2E-01)	108.24±24.59<br>90.04±21.30<br>-16.8% (6E-01)	11.31±0.52<br>9.69±0.57<br>-14.4% (6E-02)	1.28±0.10<br>1.21±0.11<br>-6.0% (6E-01)
Lithuania	136<br>87	9.94±0.88<br>7.82±0.56<br>-21.4% (5E-02)	74.39±15.14<br>54.27±6.56<br>-27.0% (2E-01)	12.13±0.61<br>8.68±0.49<br>-28.5% (4E-05)	0.99±0.06<br>1.25±0.08<br>26.5% (1E-02)
Luxembourg	44<br>17	18.25±4.23<br>8.88±1.37<br>-51.3% (3E-02)	259.81±53.54<br>199.74±57.83<br>-23.1% (5E-01)	13.27±1.35<br>8.65±0.79<br>-34.9% (7E-03)	1.38±0.15<br>1.23±0.16<br>-10.8% (5E-01)
Macedonia	19<br>28	8.37±2.03<br>12.04±1.16<br>43.8% (1E-01)	68.13±37.72<br>72.64±18.60<br>6.6% (9E-01)	10.79±1.12<br>11.11±0.75<br>2.9% (8E-01)	0.83±0.10<br>1.31±0.17<br>57.5% (2E-02)
Madagascar	17<br>12	8.12±2.03<br>11.75±2.35<br>44.7% (3E-01)	62.63±24.47<br>81.80±16.91<br>30.6% (5E-01)	12.29±1.37<br>15.00±1.83<br>22.0% (3E-01)	0.79±0.13<br>0.88±0.13<br>11.7% (6E-01)
Mexico	1,304<br>731	10.16±0.37<br>8.47±0.34<br>-16.6% (8E-04)	100.98±7.13<br>87.14±7.64<br>-13.7% (2E-01)	12.90±0.23<br>11.91±0.28<br>-7.7% (3E-03)	0.89±0.02<br>0.85±0.02<br>-4.1% (2E-01)
Morocco	262<br>77	10.59±0.66<br>8.91±0.89<br>-15.9% (1E-01)	69.65±6.03<br>80.42±18.71<br>15.5% (6E-01)	13.35±0.47<br>11.91±0.73<br>-10.8% (1E-01)	0.94±0.04<br>0.95±0.08<br>1.2% (9E-01)
Netherlands	4,536<br>2,074	23.73±0.71<br>12.52±0.34<br>-47.2% (4E-50)	466.87±15.83<br>260.14±10.68<br>-44.3% (1E-29)	13.88±0.13<br>10.14±0.14<br>-26.9% (5E-75)	1.56±0.02<br>1.45±0.02<br>-7.5% (2E-04)
New Zealand	882<br>414	18.28±1.17<br>10.42±0.57<br>-43.0% (2E-10)	309.62±23.23<br>203.14±19.06<br>-34.4% (5E-04)	16.03±0.34<br>12.02±0.40<br>-25.0% (4E-13)	1.13±0.04<br>1.04±0.04<br>-8.1% (7E-02)
Nigeria	191<br>55	10.46±0.71<br>6.69±0.68<br>-36.0% (2E-04)	63.68±5.40<br>41.55±5.99<br>-34.8% (1E-02)	14.51±0.59<br>11.98±1.18<br>-17.4% (6E-02)	0.94±0.06<br>0.79±0.06<br>-16.0% (8E-02)
Norway	1,227<br>593	16.64±0.76<br>10.84±0.61<br>-34.8% (4E-08)	301.72±19.95<br>188.24±10.53<br>-37.6% (5E-07)	14.62±0.25<br>11.76±0.28<br>-19.6% (3E-11)	1.12±0.03<br>1.13±0.04<br>0.9% (8E-01)
Pakistan	266<br>91	11.02±0.90<br>9.04±0.84<br>-17.9% (1E-01)	90.75±15.10<br>57.48±9.22<br>-36.7% (8E-02)	14.57±0.65<br>13.55±0.85<br>-7.0% (3E-01)	0.99±0.06<br>0.98±0.09<br>-1.1% (9E-01)
Peru	81<br>34	9.19±0.86<br>7.82±1.69<br>-14.8% (4E-01)	129.59±21.24<br>107.95±17.57<br>-16.7% (4E-01)	12.85±1.14<br>11.41±0.87<br>-11.2% (3E-01)	0.94±0.07<br>0.84±0.10<br>-10.1% (5E-01)
Philippines	82<br>74	10.96±2.22<br>6.77±0.70<br>-38.2% (1E-01)	220.75±75.29<br>85.58±12.44<br>-61.2% (7E-02)	10.54±0.81<br>12.03±0.77<br>14.1% (2E-01)	1.05±0.11<br>0.76±0.08<br>-27.5% (3E-02)
Poland	2,228<br>1,557	14.24±0.37<br>11.48±0.35<br>-19.4% (2E-07)	115.33±7.82<br>93.62±6.35<br>-18.8% (2E-02)	14.89±0.19<br>12.49±0.23<br>-16.1% (2E-14)	1.09±0.02<br>1.18±0.02<br>9.0% (1E-03)
Portugal	756<br>627	10.92±0.60<br>9.37±0.44<br>-14.2% (4E-02)	164.38±18.46<br>118.54±9.85<br>-27.9% (4E-02)	11.98±0.27<br>10.60±0.26<br>-11.5% (4E-04)	1.09±0.04<br>1.16±0.03<br>7.1% (1E-01)
Qatar	25<br>12	15.12±6.39<br>9.25±1.49<br>-38.8% (4E-01)	139.89±67.28<br>75.73±15.39<br>-45.9% (4E-01)	10.00±1.29<br>12.25±1.92<br>22.5% (3E-01)	1.33±0.19<br>0.86±0.10<br>-35.3% (6E-02)
Romania	365<br>399	14.88±0.98<br>11.79±0.80	76.44±8.92<br>53.39±5.11	14.59±0.64<br>12.69±0.43	1.23±0.05<br>1.19±0.05

		-20.8% (2E-02)	-30.2% (3E-02)	-13.0% (1E-02)	-3.3% (6E-01)
Russia	1,829<br>1,862	24.53±0.92<br>15.87±0.56<br>-35.3% (1E-14)	138.25±9.11<br>72.17±7.28<br>-47.8% (2E-08)	19.39±0.27<br>17.73±0.24<br>-8.5% (3E-05)	1.18±0.03<br>0.98±0.02<br>-17.1% (4E-10)
Saudi Arabia	257<br>63	13.09±0.82<br>8.71±1.01<br>-33.4% (2E-03)	107.66±9.95<br>96.51±18.22<br>-10.4% (6E-01)	12.36±0.46<br>11.33±0.96<br>-8.3% (3E-01)	1.18±0.05<br>0.94±0.07<br>-20.4% (6E-03)
Senegal	43<br>12	10.19±1.49<br>8.58±2.26<br>-15.7% (6E-01)	80.11±14.11<br>72.44±19.40<br>-9.6% (8E-01)	13.44±1.38<br>14.50±2.58<br>7.9% (7E-01)	0.94±0.10<br>0.73±0.11<br>-21.5% (2E-01)
Serbia	282<br>264	11.44±0.85<br>12.06±0.77<br>5.4% (6E-01)	59.48±5.34<br>67.10±6.96<br>12.8% (4E-01)	14.94±0.58<br>12.90±0.50<br>-13.7% (5E-03)	0.89±0.04<br>1.11±0.05<br>25.0% (7E-04)
Slovakia	318<br>220	19.36±1.58<br>16.50±1.37<br>-14.8% (2E-01)	105.51±10.19<br>95.01±10.22<br>-10.0% (5E-01)	17.47±0.60<br>14.65±0.66<br>-16.1% (4E-03)	1.11±0.04<br>1.20±0.05<br>8.5% (2E-01)
Slovenia	387<br>232	11.90±1.01<br>9.06±0.60<br>-23.8% (1E-02)	102.56±9.87<br>84.07±7.61<br>-18.0% (1E-01)	12.90±0.45<br>10.94±0.38<br>-15.2% (1E-03)	1.03±0.04<br>0.97±0.03<br>-6.1% (3E-01)
South Africa	658<br>344	18.08±1.28<br>12.30±1.08<br>-32.0% (2E-04)	238.64±23.98<br>176.86±21.29<br>-25.9% (6E-02)	15.03±0.34<br>12.54±0.45<br>-16.5% (9E-05)	1.15±0.04<br>1.09±0.05<br>-5.4% (3E-01)
Spain	5,247<br>3,617	14.47±0.35<br>11.39±0.20<br>-21.3% (2E-15)	162.43±5.04<br>136.80±3.70<br>-15.8% (1E-04)	13.43±0.10<br>11.25±0.14<br>-16.2% (5E-41)	1.19±0.02<br>1.25±0.02<br>5.1% (7E-03)
Sri Lanka	24<br>21	10.12±1.90<br>9.95±2.29<br>-1.7% (1E+00)	110.84±28.31<br>78.84±23.72<br>-28.9% (4E-01)	10.58±1.24<br>10.71±1.04<br>1.2% (9E-01)	1.05±0.11<br>0.98±0.13<br>-6.5% (7E-01)
Sweden	3,265<br>1,989	20.35±0.69<br>11.52±0.37<br>-43.4% (9E-33)	429.55±18.34<br>242.37±11.85<br>-43.6% (1E-19)	14.70±0.17<br>11.29±0.16<br>-23.2% (1E-42)	1.30±0.02<br>1.17±0.02<br>-9.6% (2E-06)
Switzerland	3,376<br>1,371	20.99±0.70<br>11.86±0.40<br>-43.5% (3E-30)	463.36±15.98<br>296.22±14.52<br>-36.1% (2E-12)	13.01±0.16<br>9.79±0.15<br>-24.7% (2E-40)	1.55±0.02<br>1.40±0.03<br>-9.4% (6E-05)
Tanzania	60<br>15	9.10±1.13<br>5.80±0.70<br>-36.3% (2E-02)	143.57±21.11<br>132.75±31.41<br>-7.5% (8E-01)	12.83±0.88<br>10.20±0.93<br>-20.5% (6E-02)	0.87±0.08<br>0.78±0.13<br>-10.7% (5E-01)
Thailand	218<br>176	12.21±1.42<br>7.98±0.47<br>-34.6% (1E-02)	189.19±41.33<br>130.33±21.04<br>-31.1% (2E-01)	11.87±0.54<br>9.86±0.40<br>-16.9% (1E-03)	1.12±0.07<br>1.03±0.04<br>-8.5% (3E-01)
Tunisia	263<br>126	10.43±0.73<br>9.07±0.84<br>-13.0% (2E-01)	71.93±7.99<br>57.00±9.95<br>-20.8% (2E-01)	12.77±0.51<br>11.06±0.61<br>-13.4% (2E-02)	0.97±0.05<br>1.03±0.08<br>5.6% (6E-01)
Turkey	3,367<br>1,493	12.38±0.29<br>10.40±0.28<br>-16.0% (1E-07)	92.51±2.82<br>83.98±3.33<br>-9.2% (8E-02)	10.42±0.09<br>9.25±0.12<br>-11.2% (3E-14)	1.40±0.02<br>1.34±0.03<br>-3.9% (8E-02)
Uganda	50<br>18	7.62±0.70<br>24.33±15.81<br>219.3% (3E-01)	180.33±32.41<br>214.40±78.44<br>18.9% (7E-01)	10.04±0.95<br>11.06±1.84<br>10.1% (7E-01)	1.12±0.11<br>1.43±0.37<br>27.3% (4E-01)
Ukraine	320<br>301	19.07±1.85<br>13.89±1.56<br>-27.2% (3E-02)	71.36±8.82<br>58.45±12.16<br>-18.1% (4E-01)	17.41±0.65<br>17.45±0.64<br>0.2% (1E+00)	1.06±0.05<br>0.95±0.07<br>-11.1% (1E-01)
United Arab Emirates	88<br>23	14.17±1.90<br>7.48±0.78<br>-47.2% (1E-03)	161.11±30.26<br>54.80±9.75<br>-66.0% (1E-03)	12.65±0.68<br>9.65±1.14<br>-23.7% (2E-02)	1.29±0.12<br>1.31±0.23<br>1.1% (1E+00)
United Kingdom	14,830<br>7,738	22.91±0.37<br>13.55±0.27<br>-40.8% (2E-101)	462.65±8.01<br>310.25±8.40<br>-32.9% (4E-38)	14.48±0.09<br>11.25±0.10<br>-22.3% (1E-135)	1.48±0.01<br>1.34±0.01<br>-9.5% (4E-17)
United States	71,722<br>37,431	20.12±0.12<br>12.45±0.10	450.41±3.83<br>296.56±2.89	14.17±0.04<br>10.97±0.04	1.42±0.00<br>1.33±0.01

		-38.1% (0E+00)	-34.2% (8E-204)	-22.6% (0E+00)	-6.6% (2E-36)
Uruguay	66	10.50±1.58	102.24±19.00	16.47±1.28	0.72±0.05
	81	7.72±0.99	93.37±13.57	11.44±0.67	0.79±0.06
		-26.5% (1E-01)	-8.7% (7E-01)	-30.5% (2E-03)	8.8% (4E-01)
Uzbekistan	10	7.20±1.93		12.30±2.53	0.85±0.13
	16	13.81±4.90		17.00±2.20	0.83±0.13
		91.8% (2E-01)		38.2% (2E-01)	-1.7% (9E-01)
Venezuela	307	11.83±0.90	95.68±14.04	14.17±0.52	0.89±0.04
	212	10.25±0.83	82.95±15.24	13.45±0.50	0.89±0.04
		-13.3% (2E-01)	-13.3% (5E-01)	-5.1% (3E-01)	-0.5% (9E-01)

Table S4. Academic performance in countries. In each cell we report the average measurements of male (blue) and female (orange) scientists, with standard errors. A third row reports the gender gap in percentage and p-value in parentheses. The p-value is calculated with two-tailed Welch's t-test to detect whether two samples with unequal size and unequal variance have identical mean.

Year of career start	Population	Total productivity	Total impact	Career length	Annual productivity
1950-1959	47,847<br>7,445	29.78±0.25<br>21.77±0.44<br>-26.9% (3E-50)	619.76±5.92<br>452.61±11.31<br>-27.0% (3E-37)	20.26±0.08<br>18.77±0.20<br>-7.3% (6E-13)	1.36±0.01<br>1.24±0.01<br>-8.7% (1E-16)
1960-1969	116,328<br>19,439	23.61±0.15<br>18.94±0.27<br>-19.7% (2E-60)	445.04±3.02<br>348.78±5.22<br>-21.6% (2E-48)	16.32±0.04<br>15.75±0.10<br>-3.5% (1E-07)	1.44±0.00<br>1.34±0.01<br>-7.0% (1E-29)
1970-1979	194,606<br>44,091	17.59±0.07<br>15.52±0.12<br>-11.8% (2E-49)	317.77±1.87<br>283.57±3.31<br>-10.8% (2E-20)	13.75±0.02<br>13.81±0.06<br>0.4% (3E-01)	1.39±0.00<br>1.31±0.00<br>-5.4% (7E-38)
1980-1989	222,255<br>71,737	10.79±0.04<br>10.40±0.06<br>-3.6% (7E-09)	185.20±1.16<br>188.28±1.90<br>1.7% (2E-01)	10.95±0.02<br>11.28±0.03<br>3.0% (2E-21)	1.18±0.00<br>1.15±0.00<br>-2.5% (7E-13)
1990-1999	288,166<br>129,567	7.78±0.02<br>7.93±0.02<br>1.9% (7E-07)	127.27±0.62<br>143.13±0.94<br>12.5% (2E-44)	8.25±0.01<br>8.58±0.01<br>4.0% (1E-98)	1.20±0.00<br>1.18±0.00<br>-1.3% (1E-06)
2000+	222,964<br>137,849	6.03±0.01<br>6.18±0.01<br>2.4% (8E-18)	103.40±0.55<br>111.84±0.67<br>8.2% (1E-23)	5.32±0.00<br>5.44±0.01<br>2.2% (3E-52)	1.55±0.00<br>1.55±0.00<br>-0.2% (5E-01)

Table S5. Academic performance given career start decade. In each cell we report the average measurements of male (blue) and female (orange) scientists, with standard errors. A third row reports the gender gap in percentage and p-value in parentheses. The p-value is calculated with two-tailed Welch's t-test to detect whether two samples with unequal size and unequal variance have identical mean.

Year of career end	Population	Total productivity	Total impact	Career length	Annual productivity
1950-1959	12,788<br>2,203	5.72±0.10<br>5.15±0.15<br>-10.0% (2E-03)	141.67±3.91<br>131.86±7.43<br>-6.9% (2E-01)	6.94±0.07<br>6.39±0.13<br>-7.9% (4E-04)	1.42±0.01<br>1.47±0.02<br>3.6% (3E-02)
1960-1969	51,474<br>8,874	6.13±0.05<br>5.19±0.09<br>-15.3% (9E-24)	136.31±1.97<br>115.59±3.60<br>-15.2% (6E-08)	6.86±0.03<br>6.23±0.07<br>-9.2% (4E-17)	1.45±0.00<br>1.47±0.01<br>1.6% (5E-02)
1970-1979	100,433<br>18,517	6.56±0.04<br>5.57±0.07<br>-15.0% (1E-34)	135.54±1.42<br>117.22±2.56<br>-13.5% (8E-11)	7.03±0.02<br>6.40±0.05<br>-9.0% (2E-31)	1.42±0.00<br>1.44±0.01<br>1.6% (5E-03)
1980-1989	164,428<br>42,738	8.84±0.04<br>6.82±0.05<br>-22.8% (4E-188)	169.60±1.25<br>127.66±1.71<br>-24.7% (8E-84)	8.78±0.02<br>7.23±0.04<br>-17.6% (0E+00)	1.28±0.00<br>1.33±0.00<br>3.5% (2E-17)
1990-1999	235,049<br>73,942	12.99±0.05<br>8.74±0.05<br>-32.7% (0E+00)	238.59±1.56<br>154.39±1.84<br>-35.3% (3E-304)	11.20±0.02<br>8.80±0.03<br>-21.4% (0E+00)	1.24±0.00<br>1.23±0.00<br>-0.3% (3E-01)
2000-2009	483,433<br>234,219	15.73±0.05<br>10.18±0.04<br>-35.3% (0E+00)	281.08±1.06<br>185.88±1.02<br>-33.9% (0E+00)	12.17±0.02<br>9.51±0.02<br>-21.8% (0E+00)	1.37±0.00<br>1.35±0.00<br>-1.2% (5E-10)
2010+	62,589<br>32,315	21.30±0.14<br>14.01±0.12<br>-34.2% (0E+00)	389.34±3.77<br>266.28±3.01<br>-31.6% (4E-143)	15.92±0.04<br>13.21±0.05<br>-17.0% (0E+00)	1.31±0.01<br>1.18±0.01<br>-9.9% (2E-65)

Table S6. Academic performance given career end decade. In each cell we report the average measurements of male (blue) and female (orange) scientists, with standard errors. A third row reports the gender gap in percentage and p-value in parentheses. The p-value is calculated with two-tailed Welch's t-test to detect whether two samples with unequal size and unequal variance have identical mean.

Institute rank	Population	Total productivity	Total impact	Career length	Annual productivity
1-19	545<br>221	29.57±0.39<br>17.92±0.40<br>-39.4% (2E-117)	756.34±9.86<br>500.26±12.69<br>-33.9% (9E-63)	15.25±0.08<br>12.02±0.11<br>-21.2% (3E-110)	1.76±0.01<br>1.55±0.01<br>-11.8% (2E-30)
20-48	280<br>108	27.09±0.29<br>16.61±0.35<br>-38.7% (2E-107)	544.44±7.80<br>357.64±8.66<br>-34.3% (2E-54)	15.02±0.09<br>11.53±0.11<br>-23.2% (1E-143)	1.65±0.01<br>1.50±0.01<br>-9.2% (1E-19)
49-86	913<br>275	27.56±0.24<br>15.92±0.31<br>-42.2% (4E-151)	537.63±6.52<br>320.17±7.30<br>-40.4% (1E-87)	15.40±0.09<br>11.43±0.12<br>-25.7% (1E-193)	1.64±0.01<br>1.49±0.01<br>-9.2% (2E-20)
87-120	2,367<br>769	26.22±0.33<br>15.41±0.32<br>-41.2% (4E-127)	496.68±7.70<br>293.95±8.26<br>-40.8% (1E-78)	14.97±0.08<br>11.28±0.12<br>-24.7% (2E-161)	1.63±0.01<br>1.48±0.01<br>-9.4% (3E-20)
121-167	1,808<br>682	23.99±0.26<br>14.50±0.27<br>-39.6% (7E-138)	449.82±6.83<br>278.99±6.99<br>-38.0% (7E-71)	14.41±0.08<br>11.02±0.08<br>-23.5% (3E-162)	1.58±0.01<br>1.48±0.01<br>-6.7% (2E-11)
168-200	0<br>0	23.56±0.40<br>15.18±0.39<br>-35.6% (1E-53)	386.04±8.66<br>234.18±8.01<br>-39.3% (3E-37)	14.48±0.11<br>11.76±0.14<br>-18.8% (2E-49)	1.55±0.01<br>1.42±0.02<br>-8.1% (2E-09)
201-250	12,350<br>4,467	24.73±0.36<br>15.99±0.42<br>-35.3% (9E-57)	433.03±8.30<br>279.65±8.75<br>-35.4% (2E-32)	15.21±0.11<br>12.15±0.14<br>-20.2% (3E-66)	1.53±0.01<br>1.40±0.02<br>-8.7% (1E-11)
251-300	16,817<br>4,913	20.91±0.26<br>13.73±0.29<br>-34.3% (9E-63)	325.41±6.13<br>229.80±7.61<br>-29.4% (5E-22)	14.09±0.08<br>11.27±0.12<br>-20.0% (4E-74)	1.47±0.01<br>1.37±0.01<br>-6.7% (3E-08)
301-350	11,803<br>4,259	21.65±0.37<br>14.61±0.34<br>-32.5% (2E-47)	383.58±8.29<br>266.23±9.94<br>-30.6% (2E-20)	14.71±0.11<br>11.69±0.15<br>-20.5% (1E-63)	1.43±0.01<br>1.38±0.01<br>-3.3% (1E-02)
351-400	7,291<br>2,491	20.81±0.44<br>13.01±0.33<br>-37.5% (6E-42)	344.03±9.54<br>204.37±9.77<br>-40.6% (3E-25)	14.04±0.15<br>10.58±0.16<br>-24.7% (5E-61)	1.46±0.01<br>1.43±0.02<br>-2.0% (2E-01)
401-500	11,893<br>4,135	19.16±0.30<br>13.23±0.34<br>-31.0% (6E-47)	264.21±5.87<br>171.65±6.16<br>-35.0% (4E-27)	14.08±0.10<br>11.69±0.14<br>-17.0% (2E-43)	1.37±0.01<br>1.34±0.02<br>-2.3% (1E-01)
501-600	7,707<br>2,692	15.61±0.35<br>11.29±0.34<br>-27.7% (3E-20)	215.82±6.48<br>142.89±6.85<br>-33.8% (2E-14)	12.70±0.12<br>10.68±0.15<br>-15.9% (4E-23)	1.31±0.01<br>1.29±0.02<br>-1.5% (4E-01)
601-800	13,674<br>4,556	15.45±0.23<br>11.68±0.27<br>-24.4% (7E-26)	175.96±4.71<br>122.98±4.84<br>-30.1% (4E-18)	13.12±0.10<br>11.43±0.13<br>-12.9% (3E-26)	1.25±0.01<br>1.23±0.01<br>-1.6% (2E-01)
801-1000	8,151<br>2,540	13.50±0.25<br>10.67±0.33<br>-21.0% (6E-13)	125.75±4.44<br>95.16±4.79<br>-24.3% (2E-06)	12.53±0.10<br>11.02±0.14<br>-12.0% (7E-14)	1.21±0.01<br>1.22±0.02<br>0.2% (9E-01)
1001+	6,338<br>2,181	12.79±0.27<br>10.84±0.37<br>-15.3% (2E-05)	105.51±3.96<br>79.00±3.40<br>-25.1% (5E-06)	12.51±0.13<br>11.37±0.17<br>-9.1% (2E-07)	1.18±0.01<br>1.19±0.02<br>0.8% (7E-01)

Table S7. Academic performance given primary affiliation rank. In each cell we report the average measurements of male (blue) and female (orange) scientists, with standard errors. A third row reports the gender gap in percentage and p-value in parentheses. The p-value is calculated with two-tailed Welch's t-test to detect whether two samples with unequal size and unequal variance have identical mean.

Number of collaborators	Population	Total productivity	Total impact	Career length	Annual productivity
0	103,414<br>23,317	3.04±0.01<br>2.92±0.02<br>-3.8% (1E-10)	27.76±0.18<br>26.91±0.33<br>-3.1% (1E-02)	6.77±0.02<br>6.20±0.03<br>-8.3% (2E-38)	1.00±0.00<br>1.00±0.01<br>-0.2% (8E-01)
1	171,648<br>47,362	3.26±0.00<br>3.20±0.01<br>-2.1% (2E-09)	31.07±0.16<br>30.91±0.29<br>-0.5% (6E-01)	5.96±0.01<br>5.76±0.02<br>-3.3% (1E-12)	1.13±0.00<br>1.11±0.00<br>-1.9% (3E-07)
2	122,155<br>39,276	4.03±0.01<br>3.76±0.01<br>-6.8% (3E-73)	43.54±0.27<br>41.65±0.36<br>-4.4% (2E-05)	6.91±0.02<br>6.24±0.03<br>-9.6% (3E-92)	1.13±0.00<br>1.14±0.00<br>0.8% (6E-02)
3	90,310<br>32,467	4.82±0.01<br>4.35±0.02<br>-9.7% (7E-118)	57.32±0.38<br>52.36±0.50<br>-8.7% (3E-15)	7.77±0.02<br>6.81±0.04<br>-12.3% (2E-131)	1.14±0.00<br>1.15±0.00<br>1.7% (4E-04)
4	70,268<br>27,939	5.59±0.02<br>4.90±0.02<br>-12.4% (4E-160)	70.55±0.50<br>62.91±0.74<br>-10.8% (5E-19)	8.54±0.03<br>7.34±0.04<br>-14.1% (3E-154)	1.14±0.00<br>1.16±0.00<br>1.5% (5E-03)
5	56,786<br>24,045	6.28±0.02<br>5.40±0.03<br>-14.0% (7E-174)	83.83±0.61<br>72.66±0.73<br>-13.3% (2E-33)	9.20±0.03<br>7.82±0.04<br>-15.0% (2E-155)	1.15±0.00<br>1.17±0.01<br>1.8% (2E-03)
6-7	86,535<br>38,789	7.38±0.02<br>6.20±0.02<br>-16.1% (0E+00)	103.37±0.60<br>87.59±0.69<br>-15.3% (1E-63)	10.05±0.03<br>8.31±0.03<br>-17.2% (0E+00)	1.18±0.00<br>1.21±0.00<br>2.2% (1E-06)
8-9	63,253<br>30,411	8.80±0.03<br>7.15±0.03<br>-18.7% (0E+00)	132.37±0.69<br>107.69±0.85<br>-18.6% (2E-85)	10.97±0.03<br>8.91±0.03<br>-18.8% (0E+00)	1.22±0.00<br>1.25±0.01<br>2.5% (9E-07)
10-11	48,674<br>23,894	10.27±0.04<br>8.07±0.04<br>-21.4% (0E+00)	161.50±1.19<br>128.05±1.34<br>-20.7% (2E-81)	11.76±0.04<br>9.39±0.04<br>-20.2% (0E+00)	1.27±0.00<br>1.30±0.01<br>1.9% (1E-03)
12-15	68,200<br>33,922	12.54±0.04<br>9.55±0.04<br>-23.9% (0E+00)	209.12±1.18<br>162.93±1.27<br>-22.1% (4E-147)	13.02±0.04<br>10.17±0.04<br>-21.9% (0E+00)	1.35±0.00<br>1.37±0.00<br>1.7% (6E-04)
16-19	45,216<br>22,043	15.95±0.07<br>11.81±0.06<br>-26.0% (0E+00)	278.49±1.76<br>213.19±2.00<br>-23.5% (1E-118)	14.58±0.05<br>11.24±0.05<br>-22.9% (0E+00)	1.45±0.01<br>1.47±0.01<br>1.4% (1E-02)
20-29	64,979<br>30,119	21.67±0.06<br>15.72±0.07<br>-27.4% (0E+00)	397.25±2.19<br>297.71±2.40<br>-25.1% (7E-220)	16.82±0.04<br>12.89±0.05<br>-23.4% (0E+00)	1.61±0.00<br>1.61±0.01<br>0.5% (3E-01)
30-49	54,788<br>21,541	34.94±0.10<br>24.14±0.12<br>-30.9% (0E+00)	689.25±3.13<br>490.24±4.21<br>-28.9% (2E-297)	21.01±0.05<br>15.96±0.06<br>-24.0% (0E+00)	1.90±0.01<br>1.86±0.01<br>-2.1% (2E-05)
50-3999	63,966<br>17,679	83.78±0.25<br>52.70±0.39<br>-37.1% (0E+00)	1,813.84±7.20<br>1,272.44±10.33<br>-29.8% (0E+00)	28.15±0.05<br>21.74±0.09<br>-22.8% (0E+00)	2.98±0.01<br>2.49±0.01<br>-16.4% (8E-220)
4000+	2<br>4	363.50±7.28<br>289.50±25.84<br>-20.4% (9E-02)	8,400.92±1,080.73<br>6,536.32±1,377.85<br>-22.2% (5E-01)	47.50±3.26<br>37.50±5.69<br>-21.1% (3E-01)	7.82±0.75<br>8.37±1.06<br>7.1% (7E-01)

Table S8. Academic performance given number of unique collaborators. In each cell we report the average measurements of male (blue) and female (orange) scientists, with standard errors. A third row reports the gender gap in percentage and p-value in parentheses. The p-value is calculated with two-tailed Welch's t-test to detect whether two samples with unequal size and unequal variance have identical mean.

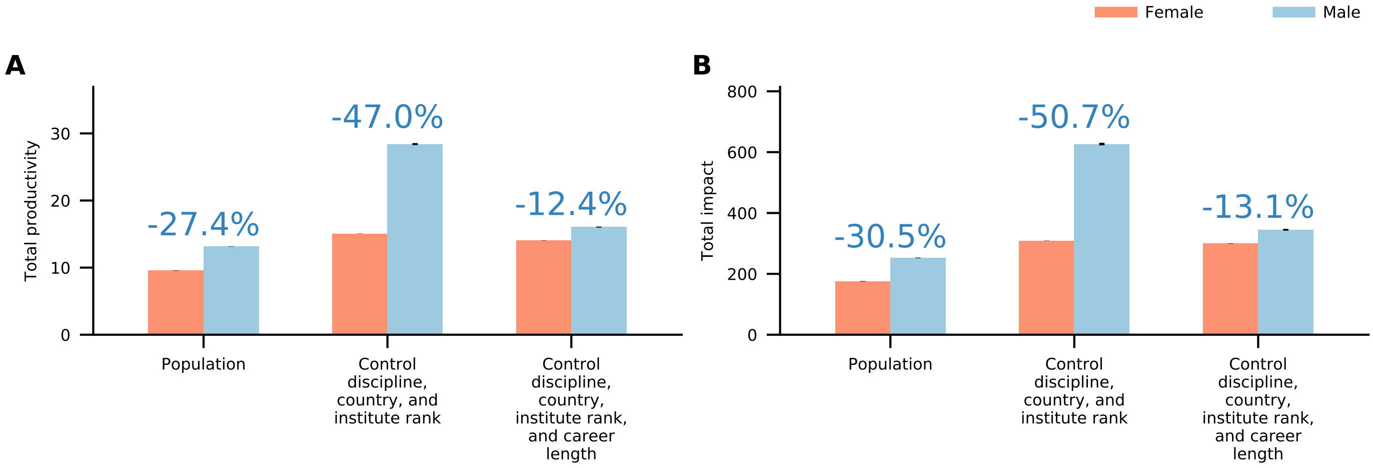

Fig. S1. Matched samples with additional constraints. The gender gap in **A**, productivity and **B**, impact when controlling for the discipline, country and affiliation rank, and the career length.

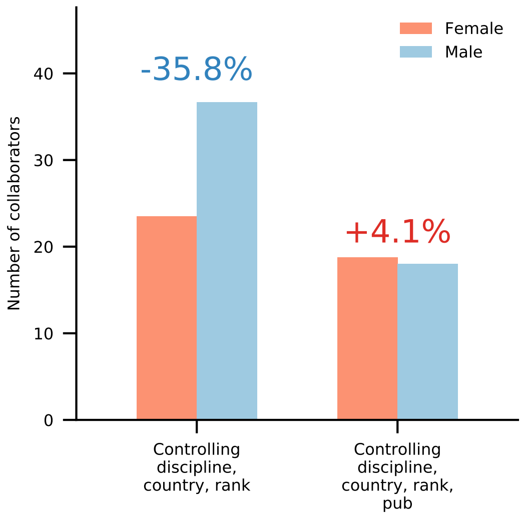

Fig. S2. Matched samples explain the average number of collaborators. The gender gap in the number of collaborators in the matched samples when controlling for the discipline, country and affiliation rank, and when controlling for he discipline, country, affiliation rank, and number of publications.

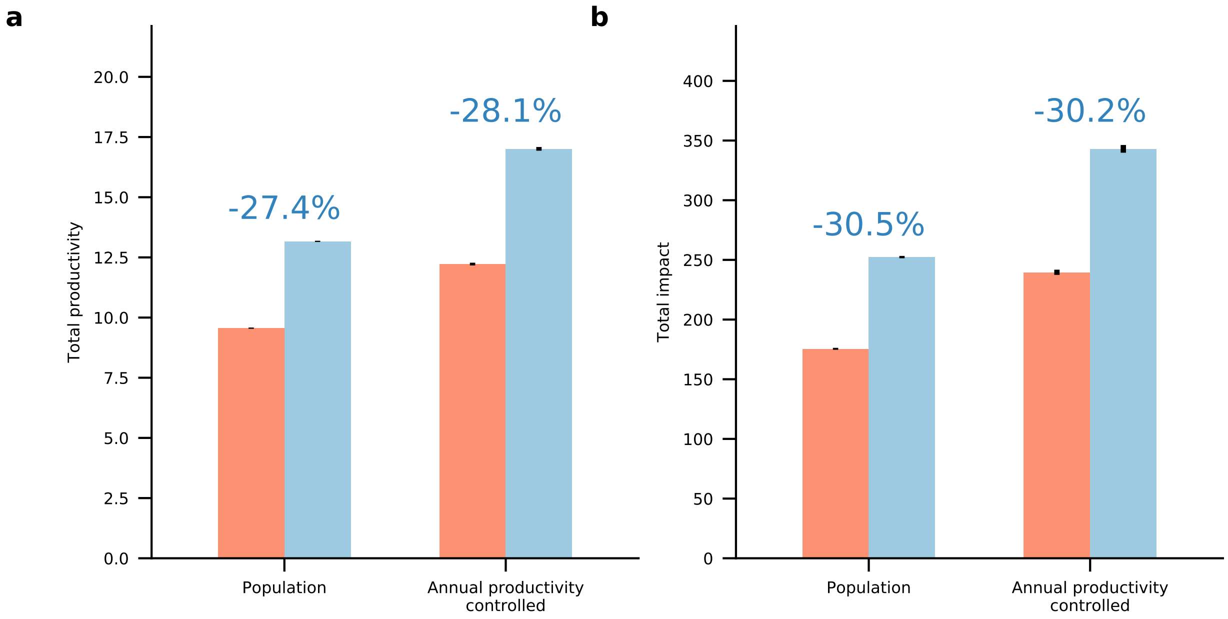

Fig. S3. Matched samples when controlling annual productivity. Gender gaps in **a** total productivity and **b** total impact, before and after we control annual productivity between genders. The correction does not reduce gender gaps in performance.

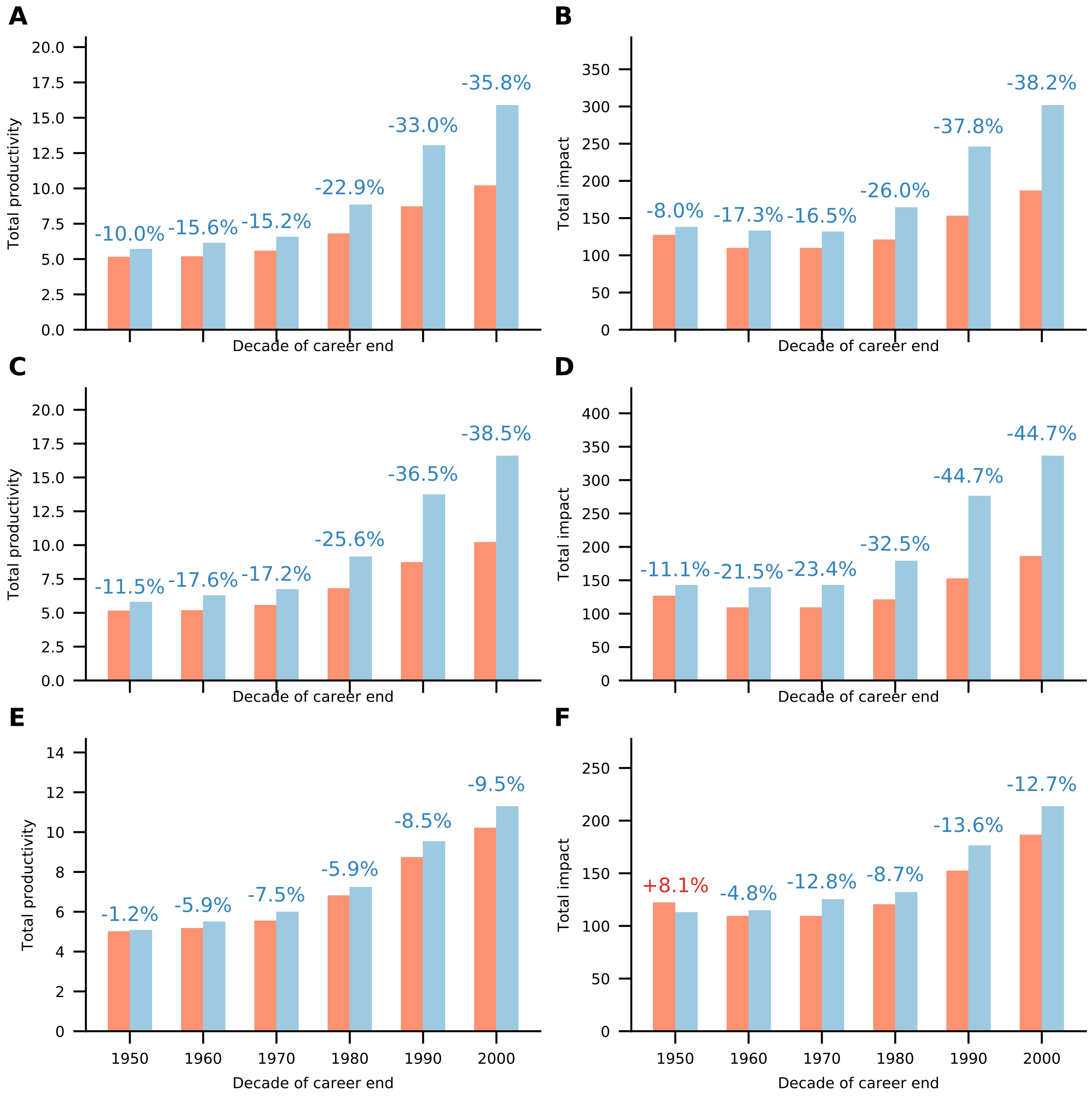

Fig. S4. Career length helps explain the increase in productivity and impact gender gaps. A,B The original gender gaps in **A**, productivity and **B**, impact conditioned on the decade in which the career ended has increased over the 60 years considered (reproduced from the main text, Figure 2**E,J**). **C,D** The gender gap in **C**, productivity and **D**, impact for the population matched by discipline. **E,F** The gender gap in **E**, productivity and **F**, impact for the population matched by discipline and career length conditioned on the decade in which the career ended. The matching experiment explains most of the growth, yet a significant fraction remains.

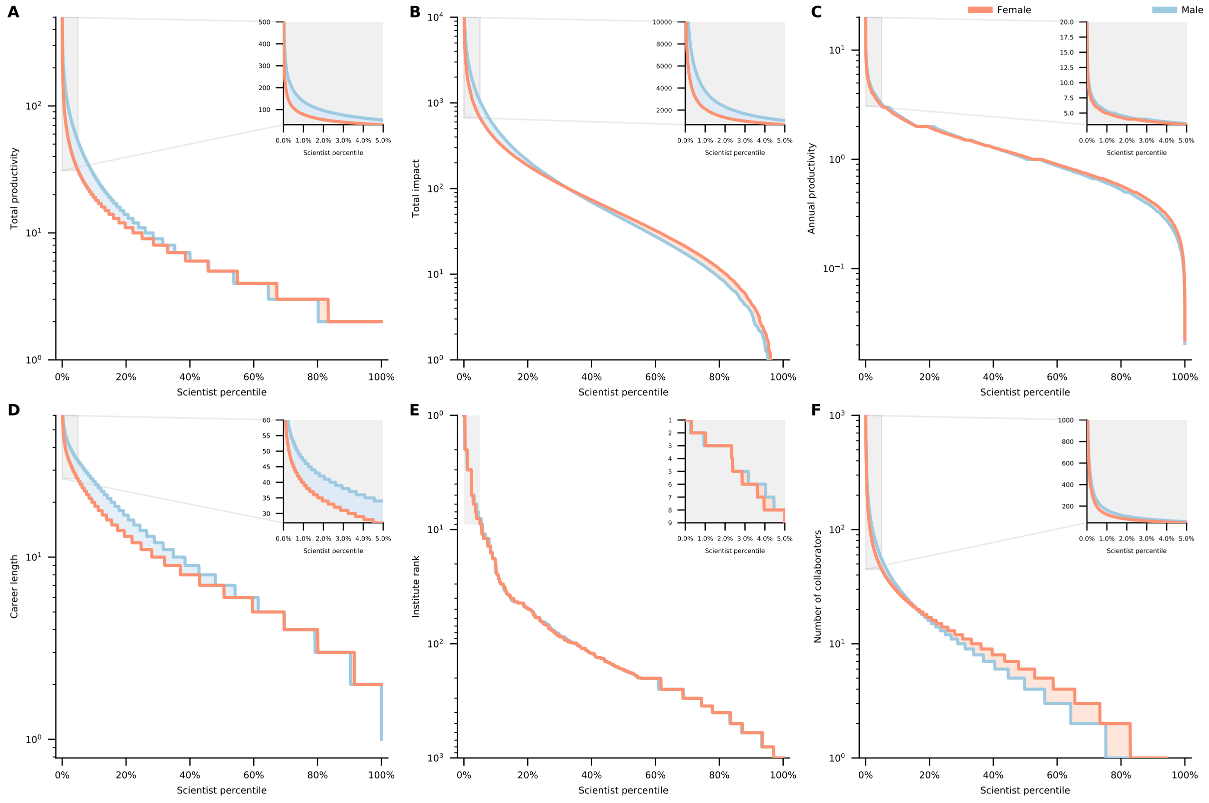

Fig. S5. Data characterization for the WoS. Distributions of **a** total productivity, **b** total impact, **c** career length, **d** annual productivity, **e** primary institute rank, **f** number of unique collaborators.

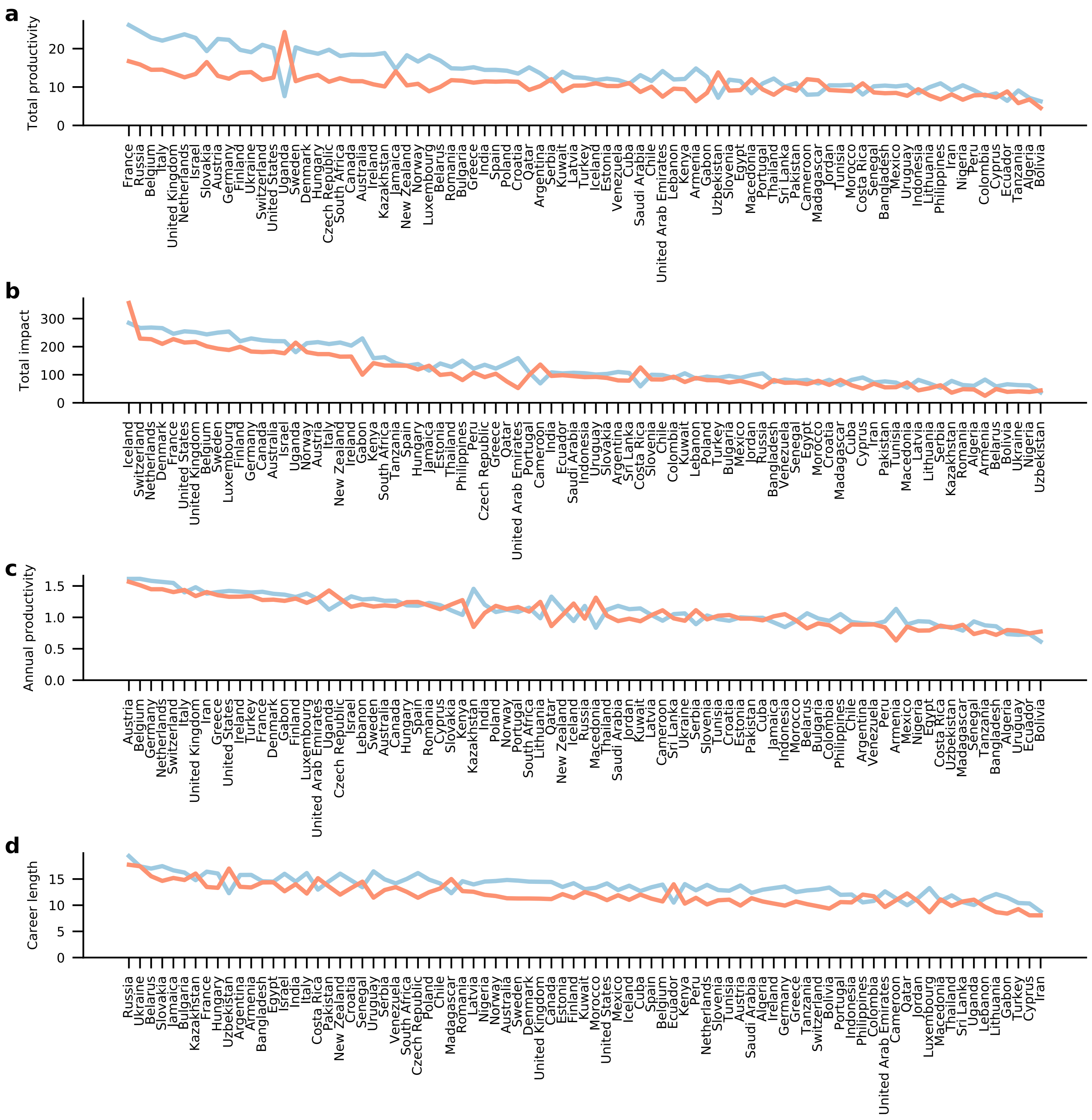

Fig. S6. The gender gap in scientific performance across countries. The average **a** total productivity, **b** total impact, **c** annual productivity, and **d** career length among all individuals in each country.

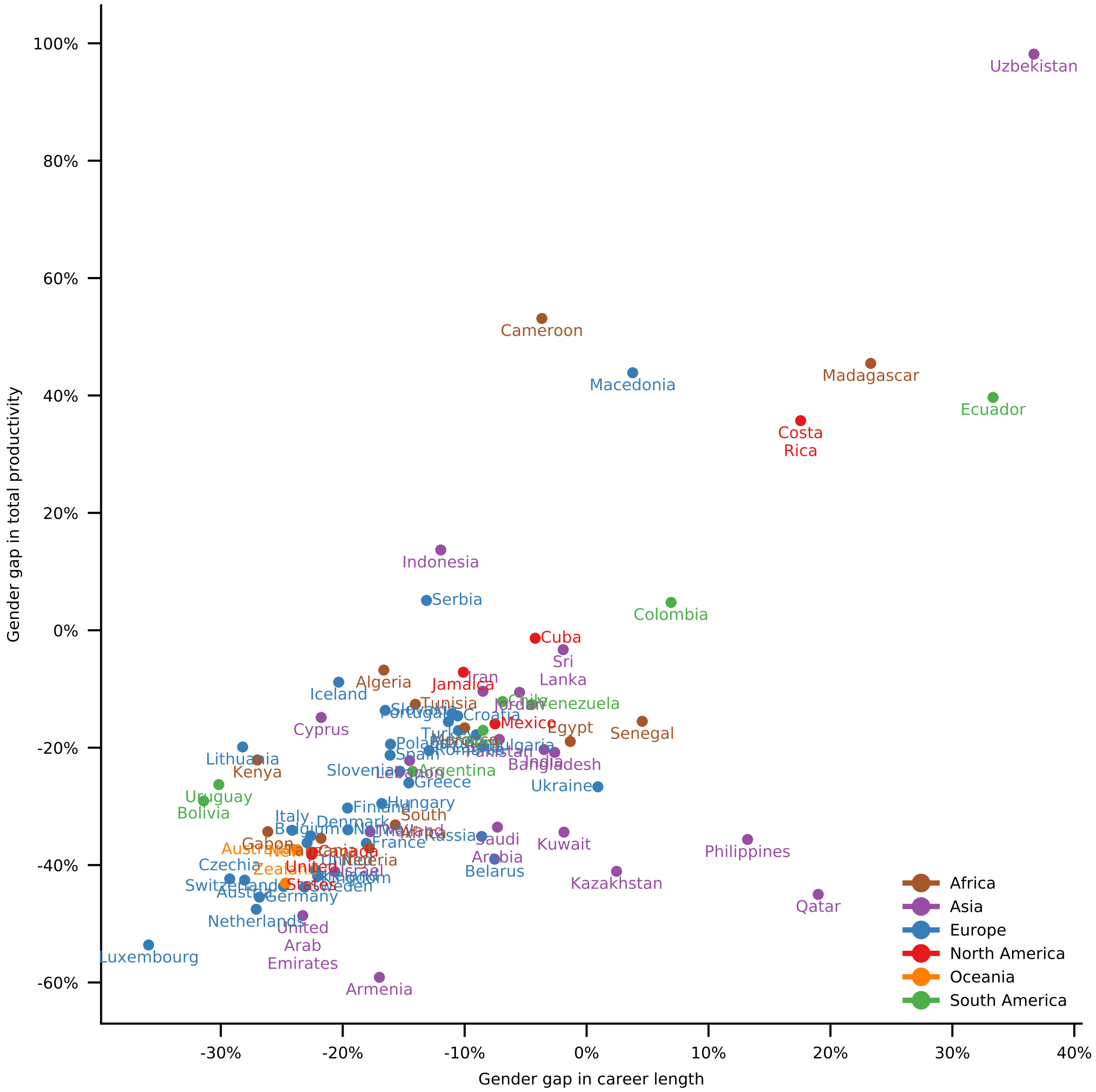

Fig. S7. The aligned gender gaps in scientific performance and career length across countries. A full version of Figure 3B (main text), demonstrating that the gender gap in career length is highly correlated with the productivity gap across countries.

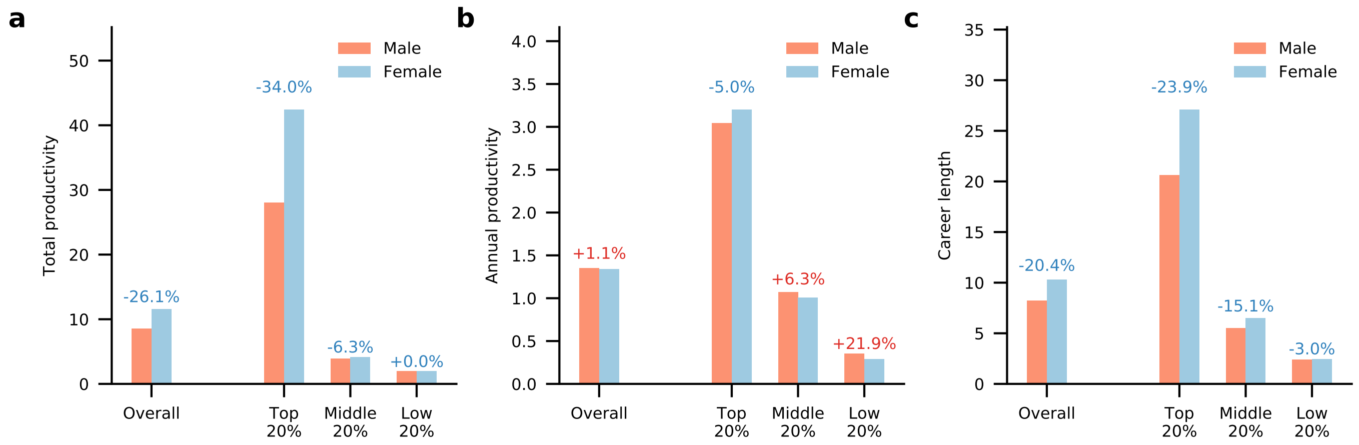

Fig. S8. The Gender Gaps in Microsoft Academic Graph. The gender gaps in **a**, total productivity, **b**, annual productivity, and **c**, career length. All three gaps mirror the results for the WoS reported in the main text.

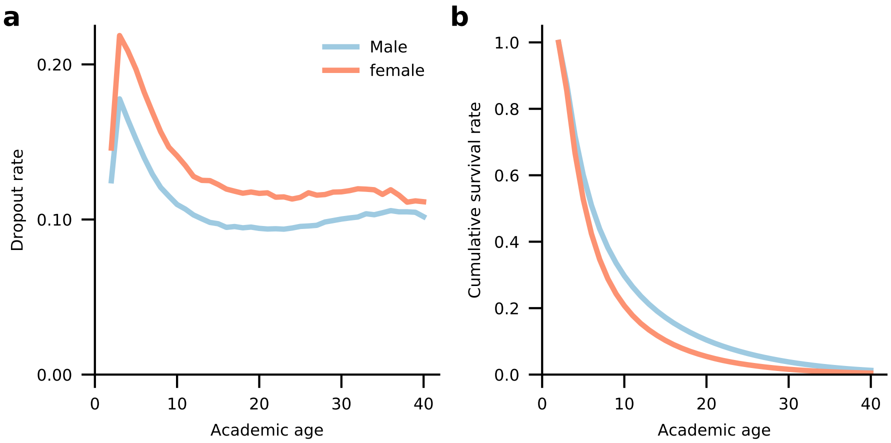

Fig. S9. Dropout and survival rates in Microsoft Academic Graph. **a**, the dropout rate of male and female scientists at each academic age. **b**, the cumulative survival rate of male and female scientists at each academic age.

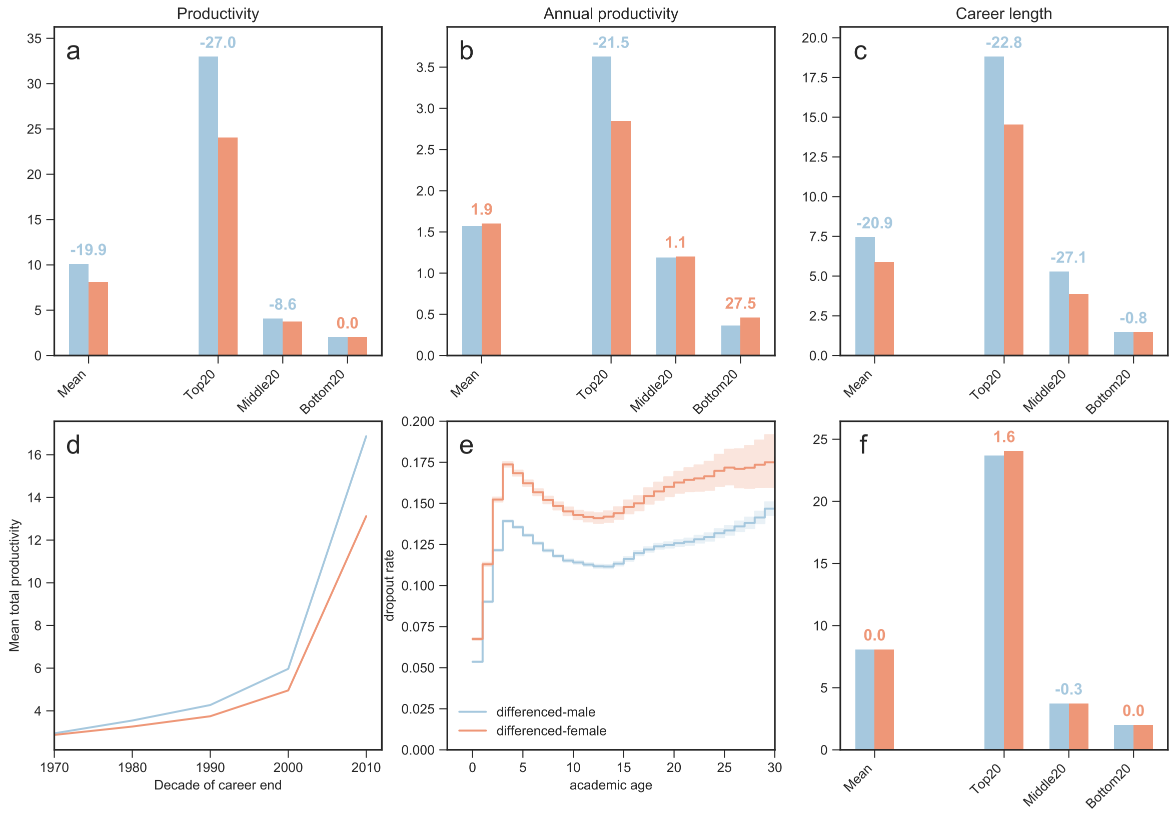

Fig. S10. The Gender Gaps in DBLP. **a**, The productivity puzzle as demonstrated by the difference in total productivity of an author during his/her career. **b**, the annual productivity is nearly identical for male and female authors. **c**, the difference in career length for male and female authors. **d**, the gender gap in productivity is growing over that last 40 years. **e**, female authors have higher dropout rate than male authors at all stages of their careers. **f**, a matching experiment eliminates the productivity gap. All conclusions qualitatively mirror the results for the WoS reported in the main text.

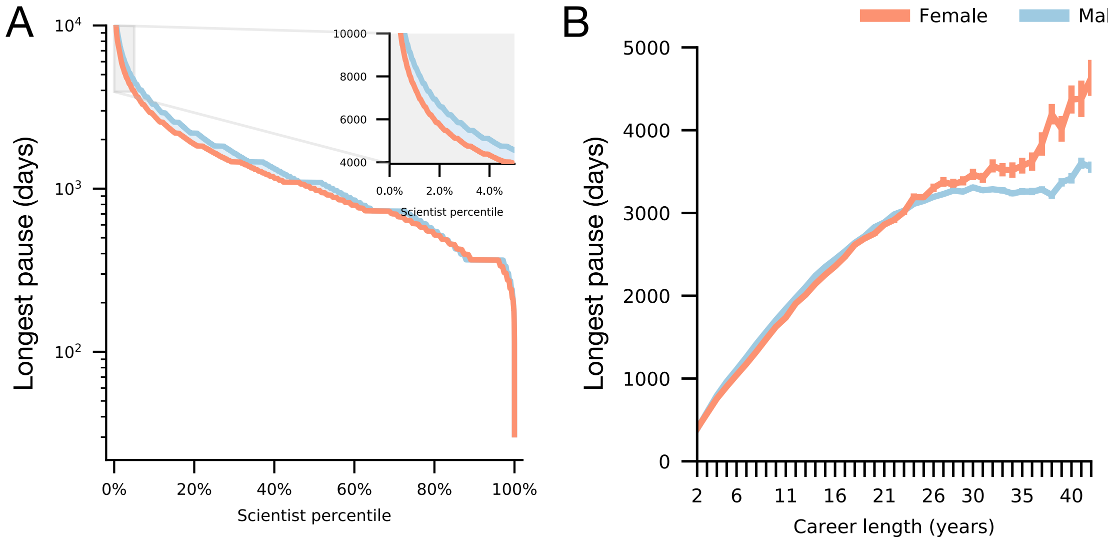

Fig. S11. Gender differences in publication pauses. **A**, The rank distribution of the longest pause in between publications (in days) for male (blue) and female (orange) authors. On average, the longest pause in a male publication career is approximately 1583 days, while the longest pause in a female publication career is only 1411 days. **B**, Male authors continue to have slightly longer pauses in between publications even when controlling for career length for careers less than 24 years (grey line), after which female authors have longer career pauses.

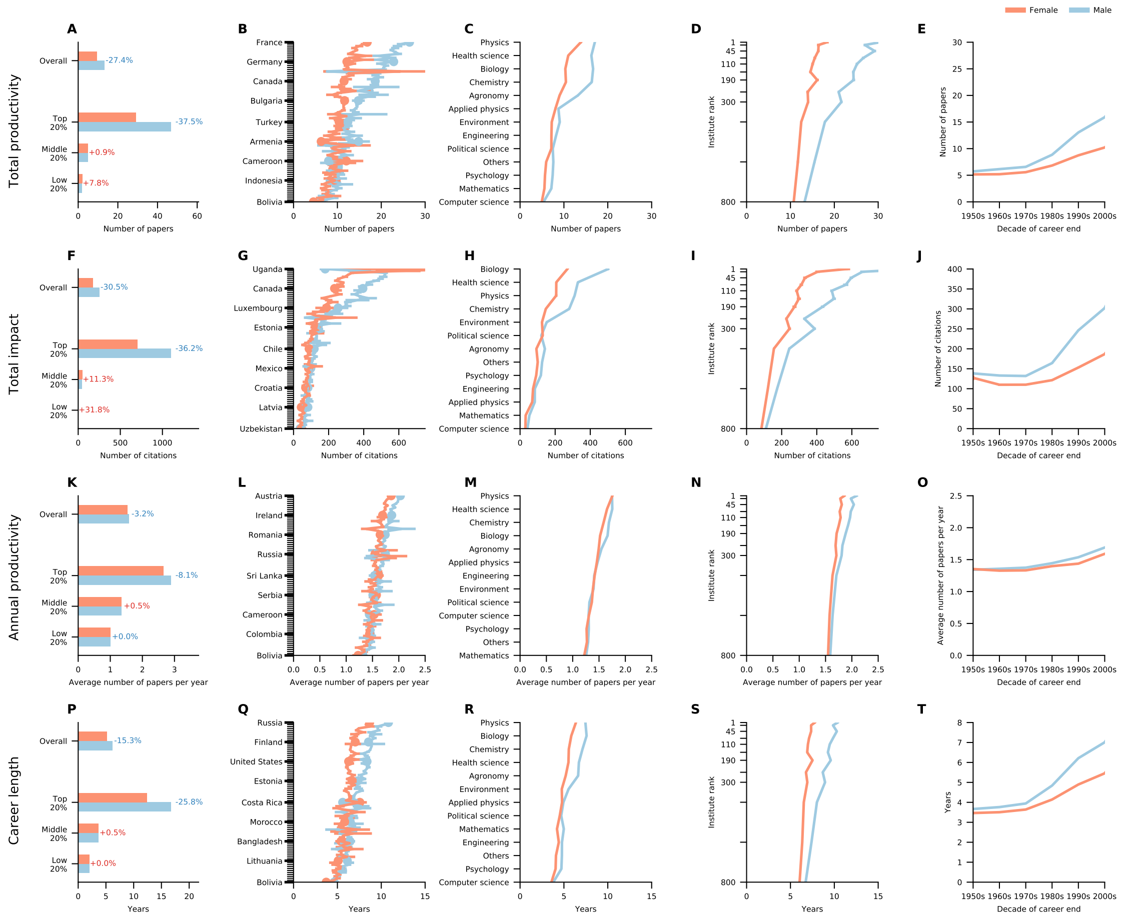

Fig. S12. Gender differences using active years. We define active years to be those years in which an author publishes as least 1 publication, while inactive careers are those years in which an author does not publish. **A-I**, The productivity and impact gender gaps reproduced from the main text, Figure 2. **K-N**, The annual productivity using active careers shows small gender differences (3% gap in overall active annual productivity). **P-S**, The active career length shows similar gender differences as the traditionally defined career length (Figure 2**P-S**).

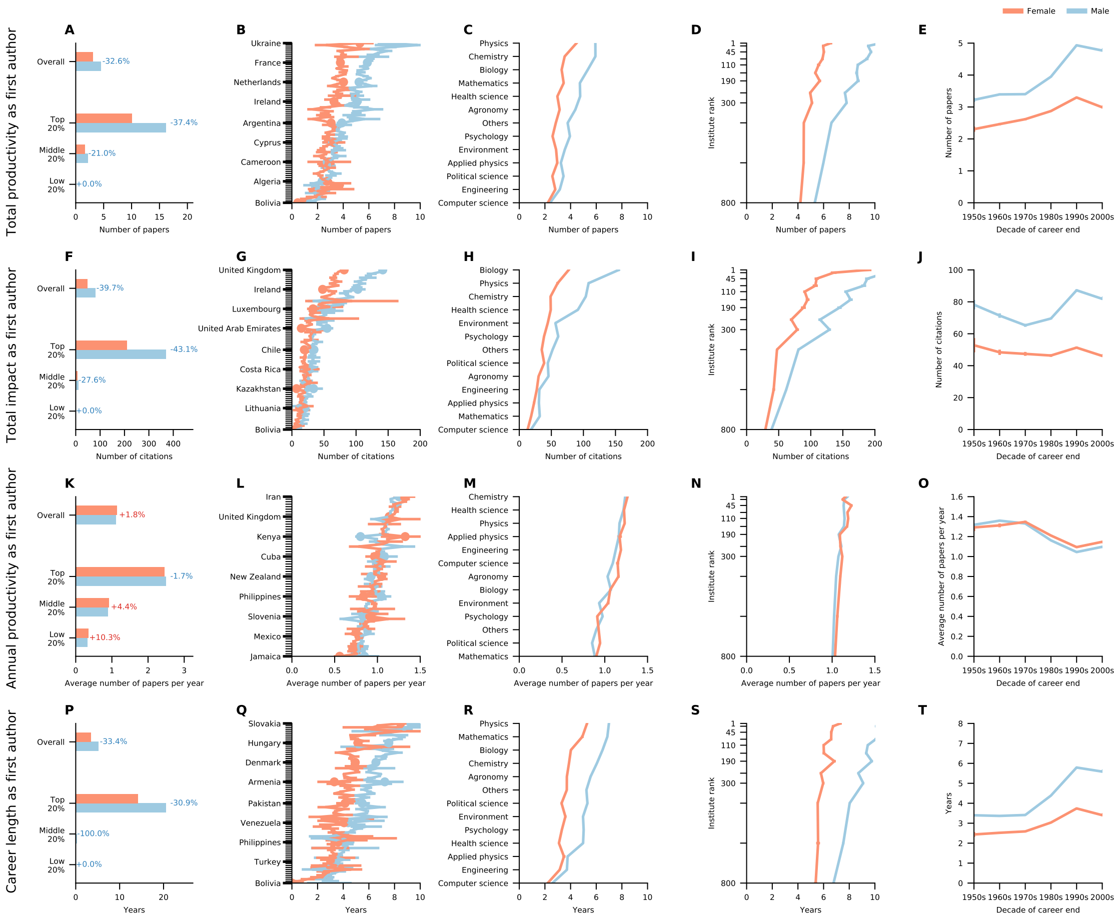

Fig. S13. Gender differences using first authorship publications. Here, publication careers are only defined for articles in which the author was the first authorship. The gender gaps are then calculated and corresponded to the same quantities as in Figure 2:. **A-E**, productivity, **F-J**, impact, **K-N**, annual productivity, and **P-S**, career length.

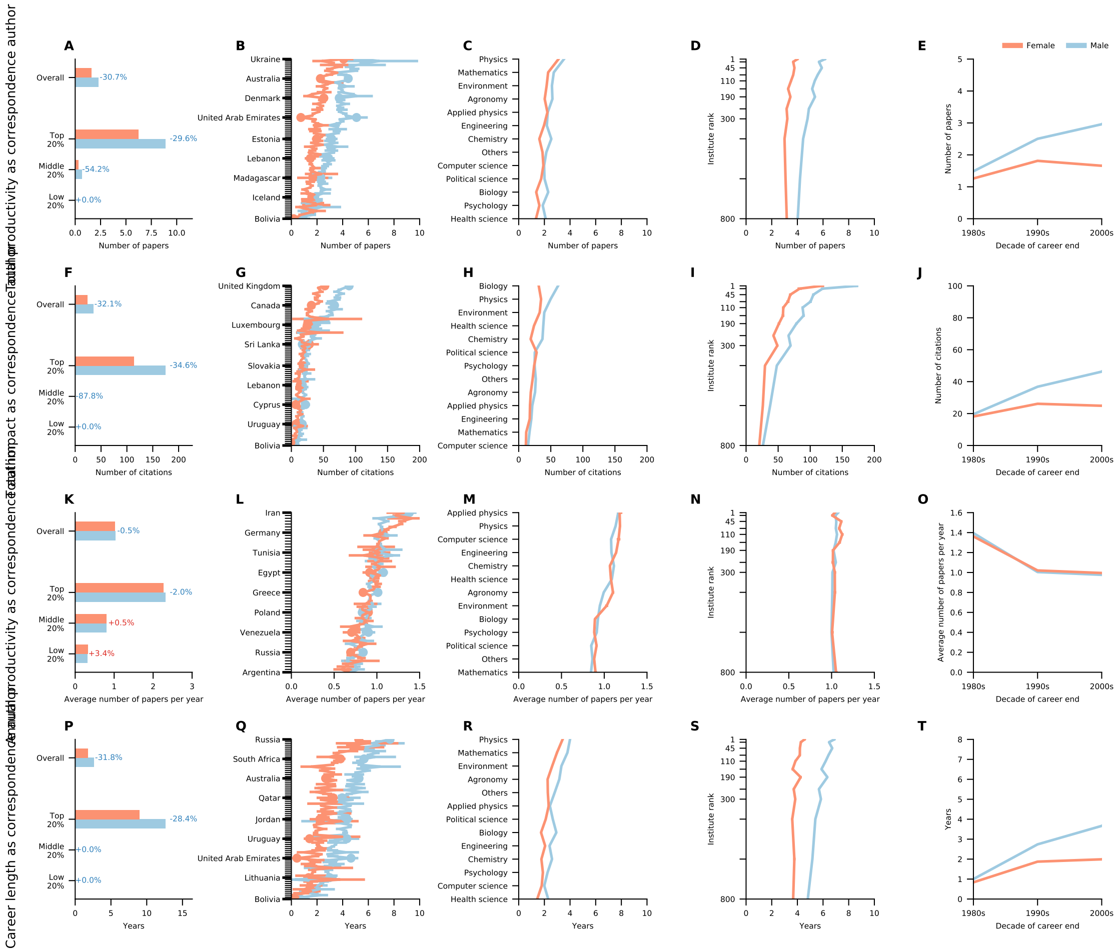

Fig. S14. Gender differences using corresponding authorship publications. Here, publication careers are only defined for articles in which the author was listed as a corresponding authorship. The gender gaps are then calculated and corresponded to the same quantities as in Figure 2:. **A-E**, productivity, **F-J**, impact, **K-N**, annual productivity, and **P-S**, career length.

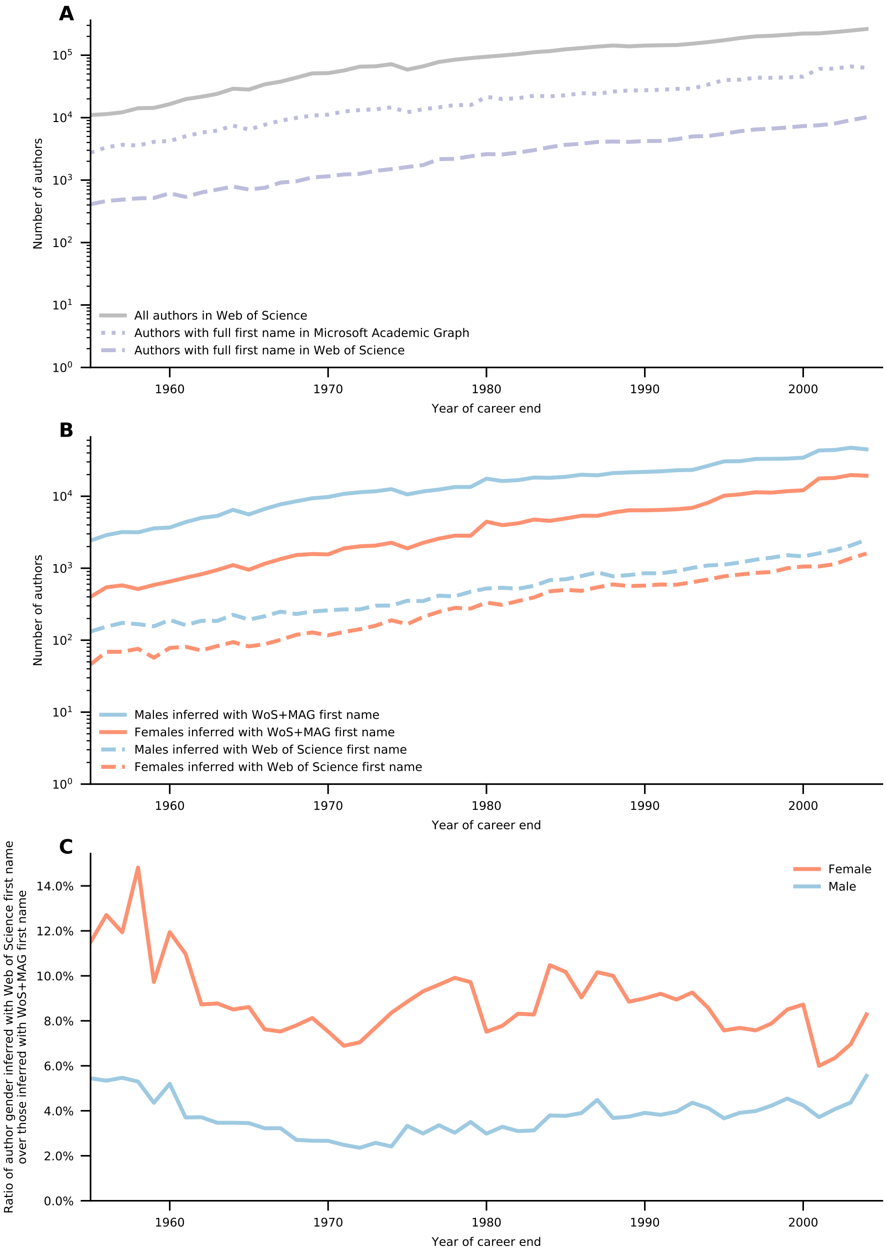

Fig. S15. Source of authors first name over time. A, The total number of authors (solid), the number of authors whose first name was inferred from the WoS (dashed), and the number of authors whose first name was inferred from the WoS supplemented by the MAG (dotted) vs the year the authors' career ended. We see no indication of temporal selection bias in the availability of first names. **B**, The number of male (blue) and female (orange) authors whose gender was inferred from the WoS names alone (dashed) and whose gender was inferred from the WoS supplemented by the MAG (solid). We see no indication of temporal bias in the identification of gender. **C**, The ratio of female authors (orange) whose gender was inferred from the WoS names alone (dashed) and whose gender was inferred from the WoS supplemented by the MAG (solid), and similar ratio for male authors (blue). We see no indication of temporal bias in the identification of gender.

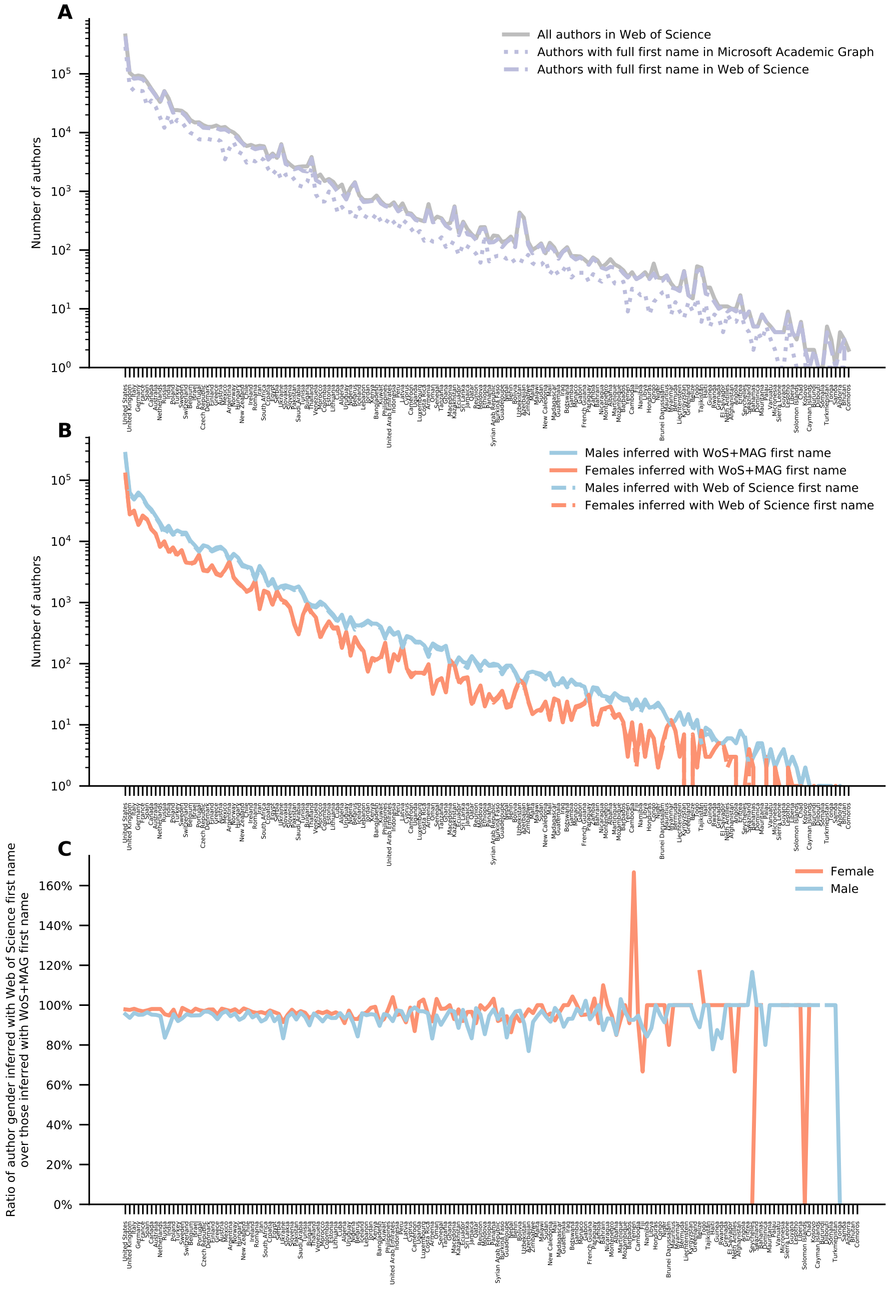

Fig. S16. Source of authors first name by country. **A**, The total number of authors (solid), the number of authors whose first name was inferred from the WoS (dashed), and the number of authors whose first name was inferred from the WoS supplemented by the MAG (dotted) vs the authors' country. We see no indication of geographic selection bias in the availability of first names. **B**, The number of male (blue) and female (orange) authors whose gender was inferred from the WoS names alone (dashed) and whose gender was inferred from the WoS supplemented by the MAG (solid). We see no indication of geographic selection bias in the identification of gender. **C**, The ratio of female authors (orange) whose gender was inferred from the WoS names alone (dashed) and whose gender was inferred from the WoS supplemented by the MAG (solid), and similar ratio for male authors (blue). We see no indication of geographic selection bias in the identification of gender.

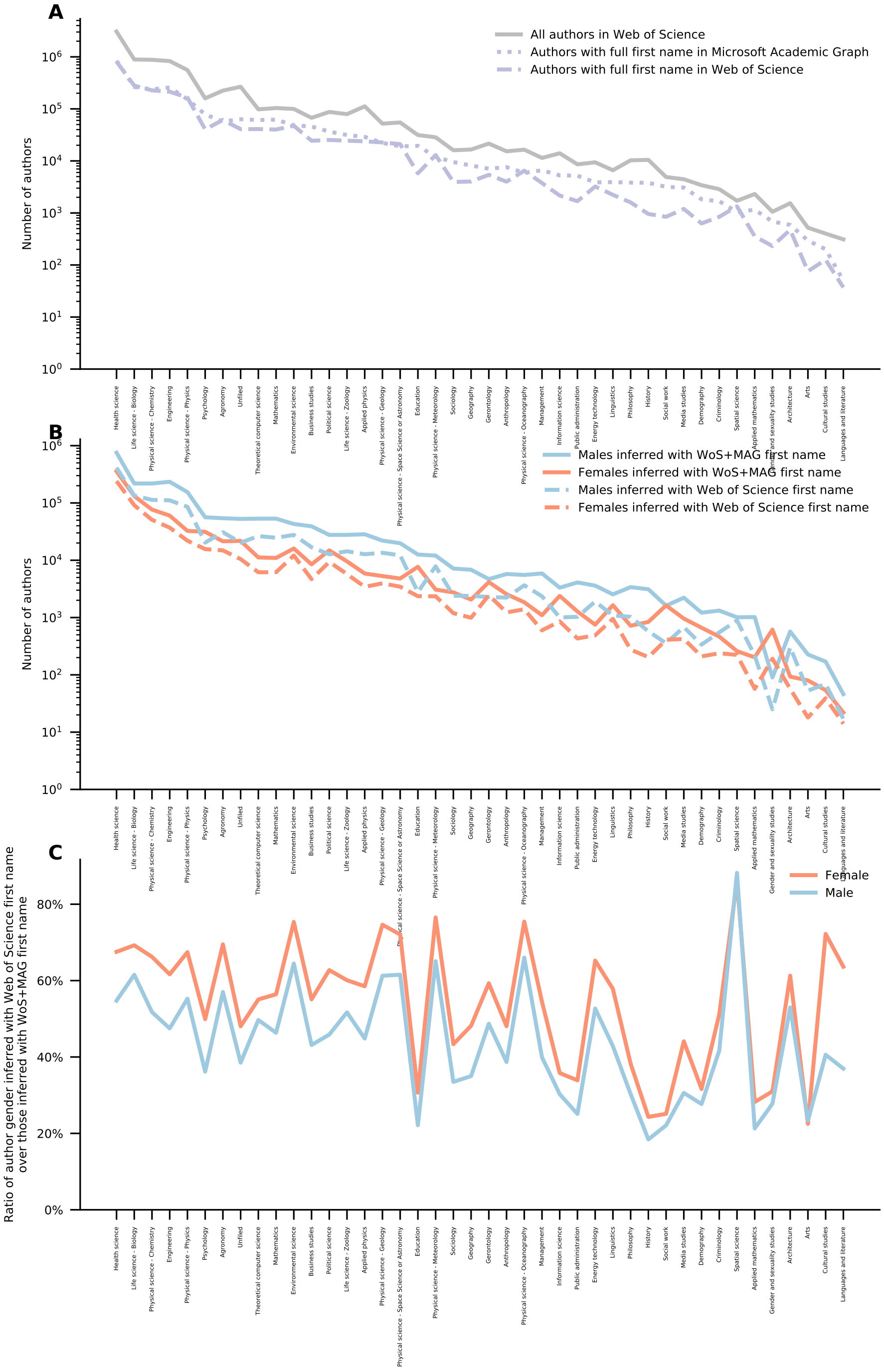

Fig. S17. Source of authors first name by discipline. **A**, The total number of authors (solid), the number of authors whose first name was inferred from the WoS (dashed), and the number of authors whose first name was inferred from the WoS supplemented by the MAG (dotted) vs the authors' discipline. We see no indication of disciplinary bias in the availability of first names. **B**, The number of male (blue) and female (orange) authors whose gender was inferred from the WoS names alone (dashed) and whose gender was inferred from the WoS supplemented by the MAG (solid). We see no indication of disciplinary bias in the identification of gender. **C**, The ratio of female authors (orange) whose gender was inferred from the WoS names alone (dashed) and whose gender was inferred from the WoS supplemented by the MAG (solid), and similar ratio for male authors (blue). We see no indication of disciplinary bias in the identification of gender.
